# Treatable childhood neuronopathy caused by mutations in riboflavin transporter RFVT2

**DOI:** 10.1093/brain/awt315

**Published:** 2013-11-15

**Authors:** A. Reghan Foley, Manoj P. Menezes, Amelie Pandraud, Michael A. Gonzalez, Ahmad Al-Odaib, Alexander J. Abrams, Kumiko Sugano, Atsushi Yonezawa, Adnan Y. Manzur, Joshua Burns, Imelda Hughes, B. Gary McCullagh, Heinz Jungbluth, Ming J. Lim, Jean-Pierre Lin, Andre Megarbane, J. Andoni Urtizberea, Ayaz H. Shah, Jayne Antony, Richard Webster, Alexander Broomfield, Joanne Ng, Ann A. Mathew, James J. O’Byrne, Eva Forman, Mariacristina Scoto, Manish Prasad, Katherine O’Brien, Simon Olpin, Marcus Oppenheim, Iain Hargreaves, John M. Land, Min X. Wang, Kevin Carpenter, Rita Horvath, Volker Straub, Monkol Lek, Wendy Gold, Michael O. Farrell, Sebastian Brandner, Rahul Phadke, Kazuo Matsubara, Michael L. McGarvey, Steven S. Scherer, Peter S. Baxter, Mary D. King, Peter Clayton, Shamima Rahman, Mary M. Reilly, Robert A. Ouvrier, John Christodoulou, Stephan Züchner, Francesco Muntoni, Henry Houlden

**Affiliations:** 1 Dubowitz Neuromuscular Centre and MRC Centre for Neuromuscular Disorders, University College London Institute of Child Health and Great Ormond Street Hospital for Children, London, WC1N 1EH, UK; 2 Institute for Neuroscience and Muscle Research, The Children’s Hospital at Westmead, Sydney, New South Wales, 2145, Australia; 3 Discipline of Paediatrics and Child Health, The University of Sydney, Sydney, New South Wales, 2006, Australia; 4 Department of Molecular Neuroscience and the MRC Centre for Neuromuscular Diseases, University College London Institute of Neurology and the Neurometabolic Unit, The National Hospital for Neurology and Neurosurgery, Queen Square, London, WC1N 3BG, UK; 5 Dr. John T. Macdonald Foundation Department of Human Genetics and Hussman Institute for Human Genomics, University of Miami Miller School of Medicine, Miami, Florida, 33136, USA; 6 Western Sydney Genetics Program, The Children’s Hospital at Westmead, Sydney, New South Wales, 2145, Australia; 7 Department of Genetics, King Faisal Specialist Hospital and Research Centre, Riyadh, 12713, Saudi Arabia; 8 Department of Clinical Pharmacology and Therapeutics, Kyoto University Hospital, Sakyo-ku, Kyoto, 606-8507, Japan; 9 Department of Paediatric Neurology, Royal Manchester Children’s Hospital, Manchester, M13 9WL, UK; 10 Department of Paediatric Neurology, Evelina Children’s Hospital, St. Thomas’ Hospital, London, SE1 7EH, UK; 11 Randall Division of Cell and Molecular Biophysics, Muscle Signalling Section, King’s College, London, WC2R 2LS, UK; 12 Clinical Neuroscience Division, Institute of Psychiatry, King’s College, London, WC2R 2LS, UK; 13 Unité de Génétique Médicale et laboratoire associe INSERM UMR S_910, Faculté de Médecine, Université Saint Joseph, Beirut, 1104 2020, Lebanon; 14 AP-HP Hôpital Marin de Hendaye, Hendaye, 64700, France; 15 Royal Aberdeen Children’s Hospital, Aberdeen, AB15 6XS, UK; 16 Metabolic Medicine Unit, Great Ormond Street Hospital for Children, London, WC1N 3JH, UK; 17 Neurology Department, Great Ormond Street Hospital for Children, London, WC1N 3JH, UK; 18 Department of Paediatric Neurology, Children’s University Hospital, Dublin, 1, Ireland; 19 Department of Paediatric Neurology, Sheffield Children’s Hospital, Sheffield, S10 2TH, UK; 20 Department of Audiology, The Children’s Hospital at Westmead, Sydney, New South Wales, 2145, Australia; 21 Clinical Chemistry, Sheffield Children’s Hospital, Sheffield, S10 2TH, UK; 22 Institute of Clinical Neurosciences, Royal Prince Alfred Hospital, Sydney, New South Wales, 2050, Australia; 23 Discipline of Genetic Medicine, The University of Sydney, Sydney, New South Wales, 2006, Australia; 24 Institute of Genetic Medicine, International Centre for Life, University of Newcastle, Newcastle upon Tyne, NE1 3BZ, UK; 25 Department of Pathology, Beaumont Hospital, Dublin, 9, Ireland; 26 Division of Neuropathology, UCL Institute of Neurology, The National Hospital for Neurology and Neurosurgery, Queen Square, London, WC1N 3BG, UK; 27 Department of Neurology, Perelman School of Medicine at the University of Pennsylvania, Philadelphia, Pennsylvania, 19104, USA; 28 Clinical and Molecular Genetics Unit, University College London Institute of Child Health and Great Ormond Street Hospital for Children, London, WC1N 1EH, UK

**Keywords:** childhood neuronopathy, Brown-Vialetto-Van Laere syndrome, riboflavin therapy, RFVT2, *SLC52A2*

## Abstract

Childhood onset motor neuron diseases or neuronopathies are a clinically heterogeneous group of disorders. A particularly severe subgroup first described in 1894, and subsequently called Brown-Vialetto-Van Laere syndrome, is characterized by progressive pontobulbar palsy, sensorineural hearing loss and respiratory insufficiency. There has been no treatment for this progressive neurodegenerative disorder, which leads to respiratory failure and usually death during childhood. We recently reported the identification of *SLC52A2*, encoding riboflavin transporter RFVT2, as a new causative gene for Brown-Vialetto-Van Laere syndrome. We used both exome and Sanger sequencing to identify *SLC52A2* mutations in patients presenting with cranial neuropathies and sensorimotor neuropathy with or without respiratory insufficiency. We undertook clinical, neurophysiological and biochemical characterization of patients with mutations in *SLC52A2,* functionally analysed the most prevalent mutations and initiated a regimen of high-dose oral riboflavin. We identified 18 patients from 13 families with compound heterozygous or homozygous mutations in *SLC52A2*. Affected individuals share a core phenotype of rapidly progressive axonal sensorimotor neuropathy (manifesting with sensory ataxia, severe weakness of the upper limbs and axial muscles with distinctly preserved strength of the lower limbs), hearing loss, optic atrophy and respiratory insufficiency. We demonstrate that *SLC52A2* mutations cause reduced riboflavin uptake and reduced riboflavin transporter protein expression, and we report the response to high-dose oral riboflavin therapy in patients with *SLC52A2* mutations, including significant and sustained clinical and biochemical improvements in two patients and preliminary clinical response data in 13 patients with associated biochemical improvements in 10 patients. The clinical and biochemical responses of this *SLC52A2*-specific cohort suggest that riboflavin supplementation can ameliorate the progression of this neurodegenerative condition, particularly when initiated soon after the onset of symptoms.

## Introduction

**Figure 1:**
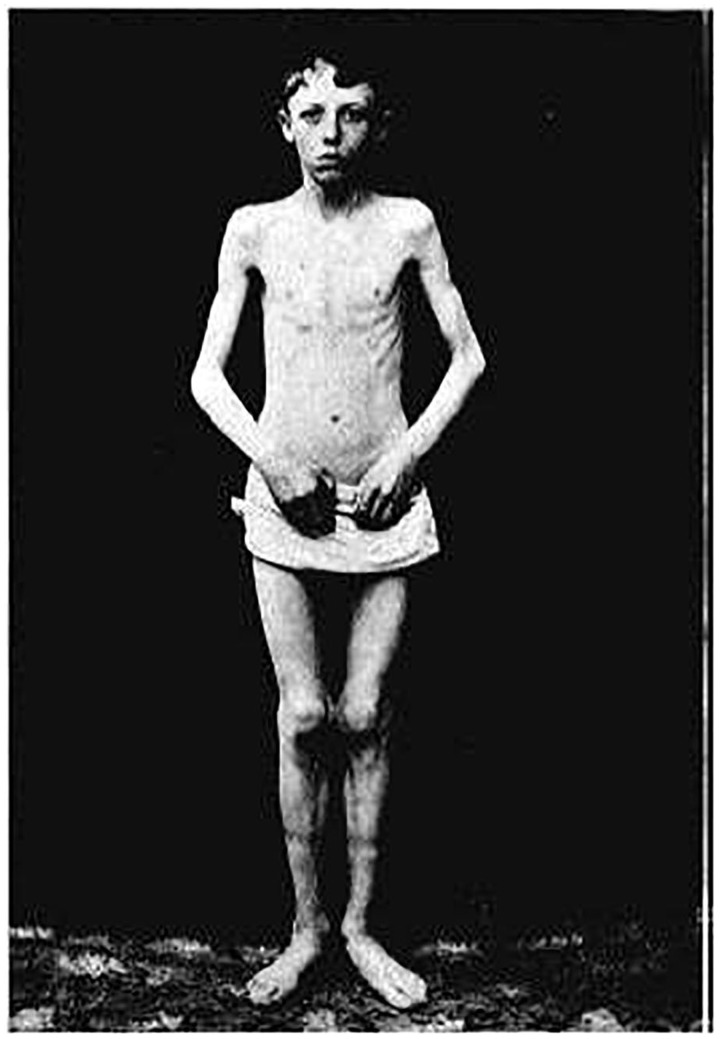
Index case from Dr Charles Henry Brown’s original report (Brown, 1894). Reproduced with permission from Kluwer Academic Publishers.

Brown-Vialetto-Van Laere syndrome (OMIM 211530) is a neurodegenerative disorder first reported in 1894 by Charles Brown as an ‘infantile’ form of amyotrophic lateral sclerosis with associated hearing loss (Brown, 1894). The male index case (Fig. 1) manifested an acute onset of bulbar weakness, hearing loss and respiratory insufficiency at age 12 years, with rapid progression of symptoms over the course of weeks. The report of three siblings with pontobulbar paralysis and associated hearing loss by Ernesto Vialetto in 1936 followed by the report of three sisters with these clinical features by M.J. Van Laere in 1966 resulted in the term Brown-Vialetto-Van Laere syndrome (Vialetto, 1936; Van Laere, 1966). Although these reports described markedly similar phenotypes, the term Brown-Vialetto-Van Laere syndrome subsequently has been assigned to a heterogeneous group of conditions, some with clear involvement of cranial nerves VII–XII and others with only minimal bulbar involvement but prominent limb weakness (Bosch *et al.*, 2012). Sensorineural deafness is a common feature of Brown-Vialetto-Van Laere syndrome and had been used to distinguish this condition from other motor neuron diseases such as Fazio-Londe disease (OMIM 211500) (McShane *et al.*, 1992), before the observation that Brown-Vialetto-Van Laere disease and Fazio-Londe disease seem to be allelic conditions that present along a phenotypic spectrum (Dipti *et al.*, 2005; Bosch *et al.*, 2011).

The recent identification of mutations in the riboflavin transporter genes *SLC52A3* (formerly *C20orf54*) (Green *et al.*, 2010) and *SLC52A2* (Johnson *et al.*, 2012) [coding for human riboflavin transporters RFVT3 (formerly RFT2) and RFVT2 (formerly RFT3), respectively] has uncovered the aetiology in a large proportion of cases with Brown-Vialetto-Van Laere syndrome. Furthermore, the recognition of abnormal acylcarnitine profiles mimicking multiple acyl-CoA dehydrogenase deficiency in patients with Brown-Vialetto-Van Laere syndrome (Bosch *et al.*, 2011) has elucidated a link between the putative function of *SLC52A3* and *SLC52A2* as riboflavin transporters and this neurodegenerative condition. In addition, a deletion in *SLC52A1*, coding for a third riboflavin transporter, RFVT1 (formerly RFT1), was reported in a case of maternal riboflavin deficiency without an associated Brown-Vialetto-Van Laere syndrome phenotype (Ho *et al.*, 2011). Here we characterize clinically, genetically and neurophysiologically 18 patients with Brown-Vialetto-Van Laere syndrome caused by mutations in the *SLC52A2* gene and report in detail the significant and sustained clinical and biochemical improvements observed in response to high-dose oral riboflavin therapy in two patients and preliminary clinical response data in 13 patients with associated biochemical response data in 10 patients.

## Materials and methods

### Study subjects

Patients were enrolled with informed consent from the patient and/or parental guardian. DNA was collected from 78 cases (72 probands and six familial cases) presenting with a phenotype of cranial neuropathies and sensorimotor neuropathy ± respiratory insufficiency. Patient DNA was collected at 21 medical centres in England (including from patients originating from Pakistan, India, Saudi Arabia, Kuwait, Iran and Turkey) and from medical centres in Wales, Scotland, Northern Ireland, Ireland, France, Belgium, The Netherlands, Greece, Malta, Russia, Lebanon, Iceland, Australia and the USA following the announcement of an ongoing molecular study at the University College London Institute of Neurology (Queen Square, London) of patients presenting with this phenotype. This study was ethically approved by the University College London Hospital, the Sydney Children’s Hospitals Network and the University of Miami Miller School of Medicine.

### Exome and Sanger sequencing

Sanger sequencing of *SLC52A2* was performed in 78 patients as described previously (Johnson *et al.*, 2012). Primer sequences are shown in the Supplementary material. Exome sequencing was performed in seven patients, with *SLC52A2* mutations confirmed by Sanger sequencing. Detailed exome sequencing methods are included in the Supplementary material. Mutations in *SLC52A1* and *SLC52A3* were excluded in all patients.

### Array-based genotyping

Haplotype analysis was carried out using single nucleotide polymorphism-based arrays or genotyping of single nucleotide polymorphisms around the *SLC52A2* gene in Patients A1–A2, A5–A7 and L1 as well as the affected members of the family described by Megarbane *et al.* (2000), to determine whether this was a shared ancestral allele, given that these patients are of Lebanese origin and all carry homozygous p.Gly306Arg *SLC52A2* mutations.

### Riboflavin uptake, SLC52A2 protein expression and RNA expression

Seven *SLC52A2* mutations [p.Trp31Ser (c.92G > C), p.Gln234X (c.700C > T), p.Ala284Asp (c.851C > A), p.Tyr305Cys (c.914A > G), p.Gly306Arg (c.916G > A), p.Leu312Pro (c.935T > C) and p.Leu339Pro (c.1016T > C)] were analysed in an *in vitro* transient expression system. ^3^H-riboflavin transport activity, protein expression and RNA expression of SLC52A2 (92G > C; W31S), SLC52A2 (700C > T; Q234X), SLC52A2 (851C > A; A284D), SLC52A2 (914A > G; Y305C), SLC52A2 (916G > A; G306R), SLC52A2 (935T > C; L312P) and SLC52A2 (1016T > C; L339P) were assessed. Detailed methods are included in the Supplementary material.

### High-dose oral riboflavin therapy

Oral riboflavin was commenced at a dose of 10 mg/kg/day and sequentially increased to 50 mg/kg/day in paediatric patients and 1500 mg/day in adult patients. We performed clinical and biochemical assessments before and after the initiation of oral riboflavin, following recommendations personally shared by Dr Annet Bosch (Department of Paediatrics, University of Amsterdam, The Netherlands) and published online (http://www.bvvlinternational.org/B2_Therapy_Protocol.html).

## Results

### *SLC52A2* mutations

*SLC52A2* mutations were found in 18 individuals: 13 probands, including one previously described individual [Patient E1; Family D (Johnson *et al.*, 2012)], and five affected family members. Eight of the individuals harbouring *SLC52A2* mutations (Patients E2–5, A1–2, I1 and L1; seven probands and one affected sibling) had been specifically selected for Sanger sequencing of *SLC52A2* after being identified as having a clinical phenotype evocative of the initial report (Johnson *et al.*, 2012). Three patients (Patients E1, E6 and E7) were part of a cohort of 63 unrelated individuals with cranial neuropathies and sensorimotor neuropathy ± respiratory insufficiency that were Sanger sequenced for mutations in *SLC52A1*, *SLC52A2* and *SLC52A3*. Seven patients (Patients A3–A7, U1 and U2; three probands and four affected siblings) had *SLC52A2* mutations found through exome sequencing.

**Figure 2:**
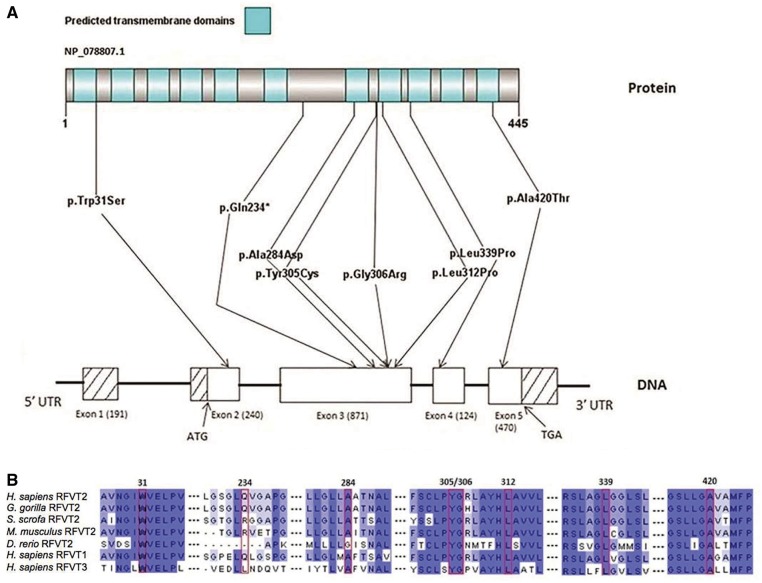
Mutations in *SLC52A2* in Brown-Vialetto-Van Laere syndrome. (**A**) Predicted transmembrane domains in RFVT2, gene structure and location of mutations identified in *SLC52A2* in this patient cohort. Reference sequence NM_024531.4. The Washington Exome Variant Server (http://evs.gs.washington.edu/EVS/), single nucleotide polymorphism database (dbSNP) (http://www.ncbi.nlm.nih.gov/snp) and 1000 Genomes Project (http://www.1000genomes.org/) databases were screened for the identified mutations. (**B**) Structural conservation of relevant amino acid residues in RFVT2 across species and in RFVT1 and RFVT3. Dark blue, medium blue and light blue colours correspond to amino acids conserved in ≥6, ≥5 or ≥3 of 7 sequences, respectively. Conservation among species of the affected amino acid residues was determined using Ensembl to retrieve the sequences and Clustal Omega software (Thompson *et al.*, 1994) for multiple sequence alignment. The Ensembl protein IDs for the RFVT2 orthologous sequences reported are ENSGGOP00000028056, ENSSSCP00000028741, ENSMUSP00000023220 and ENSDARP00000045674.

The nature and location of each *SLC52A2* mutation are shown in Fig. 2A. Seven different missense mutations and one premature stop mutation were identified. The *SLC52A2* mutations p.Gly306Arg (c.916G > A) and p.Leu339Pro (c.1016T > C) have been reported previously (Haack *et al.*, 2012; Johnson *et al.*, 2012). The other six mutations reported here are novel. All mutations were predicted as not tolerated by the SIFT prediction program and predicted as probably damaging by PolyPhen2, except for p.Ala284Asp (c.851C > A), which was predicted as possibly damaging. All mutations except the nonsense mutation p.Gln234X (c.700C > T) (only conserved in the *Gorilla gorilla* and RFVT1) and the p.Ala284Asp (c.851C > A) mutation (not conserved in *Danio rerio*) alter amino acids evolutionarily conserved from humans to *D. rerio* and are also conserved in RFVT1 and RFVT3 (Fig. 2B).

Patients A1, A2, A5, A6, A7 and L1, and the two affected members of the original Lebanese BVVL family (Megarbane *et al.*, 2000) were found to be homozygous for the p.Gly306Arg mutation and at all 11 single nucleotide polymorphisms studied, indicating that the mutation arose on a common haplotype within the Lebanese population as a founder mutation (data available on request).

### Clinical presentation

Eighteen patients (13 probands) were found to harbour either compound heterozygous or homozygous mutations in *SLC52A2*. Clinical and genetic features of these patients are listed in Table 1. Seven patients were identified in England (Patients E1–E7), seven in Australia (Patients A1–A7), two in the USA (Patients U1 and U2), one in Ireland (Patient I1) and one in Lebanon (Patient L1). Our cohort demonstrates a female predominance, with a female to male ratio of 11:7.

**Table 1:** Clinical and genetic features of patients with Brown-Vialetto-Van Laere syndrome caused by mutations in *SLC52A2* (at the time of diagnosis, before initiating oral riboflavin therapy)

Patient	E1^b^	E2	E3	E4	E5	E6	E7
*SLC52A2* mutations	Homozygous: c.[916G > A]; p. [(G306R)]	Compound heterozygous: c.[92G > C]; [935T > C] p.[(W31S)]; [(L312P)]	Compound heterozygous: c.[700C > T]; [1258G > A] p.[(Q234X)]; [(A420T)]	Compound heterozygous: c.[916G > A]; [1016T > C] p.[(G306R)]; [(L339P)]	Compound heterozygous: c.[916 G > A]; [1016T > C] p.[(G306R)]; [(L339P)]	Compound heterozygous: c.[935T > C]; [1016T > C] p.[(L312P)]; [(L339P)]	Compound heterozygous: c.[916 G > A]; [1258G > A] p.[(G306R)]; [(A420T)]
Sex	F	F	F	F	F	F	M
Ethnicity	Scottish	English and Icelandic	English	English and Scottish	Scottish	English	English
Consanguinity	Not reported	No	No	No	No	No	No
First symptom	Ataxic gait	Ataxic gait	Ataxic gait	Right-sided ptosis	UL weakness	Nystagmus	Hearing loss
Age at first symptom	1.5 yrs	1 yr	3.5 yrs	1.5 yrs	3 yrs	0.6 yr	2 yrs
Optic atrophy	Yes	Yes	Yes	Yes	NA	Yes	Yes
Sensorineural hearing loss	Yes	Yes	Yes	Yes	Yes	Yes	Yes
Sensorimotor neuropathy^a^	Yes	Yes	Yes	Yes	Yes	Yes	Yes
Distribution of weakness	UL > LL; neck extension	UL > LL; neck extension	UL > LL; neck extension	UL > LL; neck extension	UL > LL; neck extension	UL > LL; neck extension	UL > LL
Overall maximal motor function	Independent ambulation	Independent ambulation	Independent ambulation	Independent ambulation	Independent ambulation	Independent ambulation	Independent ambulation
Maximal motor function at the time of diagnosis (prior to riboflavin therapy)	Taking a few steps with head and trunk supported	Unable to sit	Walking if trunk supported	Walking if trunk supported	Taking a few steps with head and trunk supported	Taking a few steps with head and trunk supported	Taking a few steps with head and trunk supported
Respiratory function	Nocturnal NIV	Ventilator dependent	Ventilator dependent	Nocturnal NIV	Nocturnal NIV	Nocturnal NIV	Nocturnal NIV
Feeding	By mouth	By gastrostomy only	By mouth and gastrostomy	By mouth	By gastrostomy only	By mouth and gastrostomy	By mouth and gastrostomy
Age at genetic diagnosis	10 yrs	6 yrs	5 yrs	5 yrs	12 yrs	17.5 yrs	21.5 yrs

	Family 1		Family 2		Family 3			Family 4			
Patient	A1	A2	A3	A4	A5^c^	A6^c^	A7	U1	U2	I1	L1
*SLC52A2* mutations	Homozygous: c.[916G > A]; p. [(G306R)]	Homozygous: c.[916G > A]; p. [(G306R)]	Compound heterozygous: c.[916 G > A]; [1016T > C] p. [(G306R)]; [(L339P)]	Compound heterozygous: c.[916 G > A]; [1016T > C] p. [(G306R)]; [(L339P)]	Homozygous: c.[916G > A]; p. [(G306R)]	Homozygous: c.[916G > A]; p. [(G306R)]	Homozygous: c.[916G > A]; p. [(G306R)]	Compound heterozygous: c. [851C > A]; [916G > A] p.[(A284D)]; [(G306R)]	Compound heterozygous: c. [851C > A]; [916G > A] p.[(A284D)]; [(G306R)]	Compound heterozygous: c.[914A > G]; [916G > A] p.[(Y305C)]; [(G306R)]	Homozygous: c.[916G > A]; p.[(G306R)]
Sex	F	F	F	F	M	M	M	F	M	M	M
Ethnicity	Lebanese	Lebanese	English and Scottish	English and Scottish	Lebanese	Lebanese	Lebanese	Native American, Scottish, Irish, English and German	Native American, Scottish, Irish, English and German	Irish	Lebanese
Consanguinity	Yes	Yes	No	No	Yes	Yes	Yes	No	No	No	Yes
First symptom	Ataxic gait	Ataxic gait	Ataxic gait and UL weakness	Hearing loss	Ataxic gait and hearing loss	Ataxic gait and hearing loss	Ataxic gait	Respiratory failure	Vision loss	Nystagmus	Hearing loss
Age at first symptom	8 yrs	3 yrs	2 yrs	5 yrs	3 yrs	3 yrs	5 yrs	2 yrs	4 yrs	1.3 yrs	3 yrs
Optic atrophy	No	Yes	NA	Yes	Yes	Yes	Yes	Yes	Yes	Yes	NA
Sensorineural hearing loss	Yes	Yes	NA	Yes	Yes	Yes	Yes	Yes	Yes	Yes	Yes
Sensorimotor neuropathy^a^	Sensory only	Yes	Yes	Yes	Yes	Yes	Yes	Yes	Yes	Yes	Yes
Distribution of weakness	None detected	UL	UL	UL; neck extension	UL; neck extension	UL; neck extension	UL	UL > LL	UL > LL	UL > LL; neck extension	UL > LL; neck extension
Overall maximal motor function	Independent ambulation	Independent ambulation	Independent ambulation	Independent ambulation	Independent ambulation	Independent ambulation	Independent ambulation	Independent ambulation	Independent ambulation	Independent ambulation	Independent ambulation
Maximal motor function at the time of diagnosis (prior to riboflavin therapy)	Independent ambulation	Independent ambulation	Taking a few steps with head and trunk supported	Independent ambulation	Taking a few steps with head and trunk supported	Taking a few steps with head and trunk supported	Independent ambulation	Independent ambulation	Walking with a cane	Sitting	Independent ambulation
Respiratory function	Normal	Normal	Decreased; on ventilator at the time of death	Decreased; nocturnal NIV recommended	Ventilator dependent	Nocturnal NIV	Normal	Nocturnal NIV	Normal	Ventilator dependent	Normal
Feeding	By mouth	By mouth	By mouth	By mouth	By gastrostomy only	By gastrostomy only	By mouth	By mouth	By mouth	By nasogastric tube only	By mouth
Age at genetic diagnosis	10 yrs	9 yrs	Deceased (3.5 yrs)	15 yrs	16 yrs	16yrs	21 yrs	52 yrs	44 yrs	1.9 yrs	6 yrs

^a^Based on nerve conduction studies (Table 2).

^b^Proband from Family D (Johnson *et al.*, 2012).

^c^Identical twins.

F = female; M = male; LL = lower limbs; NA = not assessed (formally); NIV = non-invasive ventilation; UL = upper limbs; yr = year.

The most common presenting symptom was an ataxic gait, reported in 9 of 18 (50%) patients, secondary to a progressive sensory neuropathy. Parents reported noticing symptoms as early as age 7 months (nystagmus) and as late as age 8 years (ataxic gait). Optic atrophy was diagnosed in 14 of 15 patients (93%) after formal ophthalmologic evaluations, often prompted by evidence of nystagmus. In particular, nystagmus was the presenting symptom in Patient E6 at just 7 months of age and Patient I1 at 16 months of age. All 18 patients had symptoms of hearing loss with audiometry documenting bilateral sensorineural hearing loss. Tongue fasciculations were documented in 11 of 18 patients with evidence of a correlation between severity of fasciculations and length of time following the onset of first symptoms. No tongue fasciculations were evident in Patient I1 who started riboflavin therapy 6 months after the emergence of his first symptom, whereas tongue fasciculations along with tongue weakness and atrophy were noted in Patient U1, who was found to harbour mutations in *SLC52A2* and was initiated on riboflavin therapy 50 years after the onset of her first symptom.

**Figure 3:**
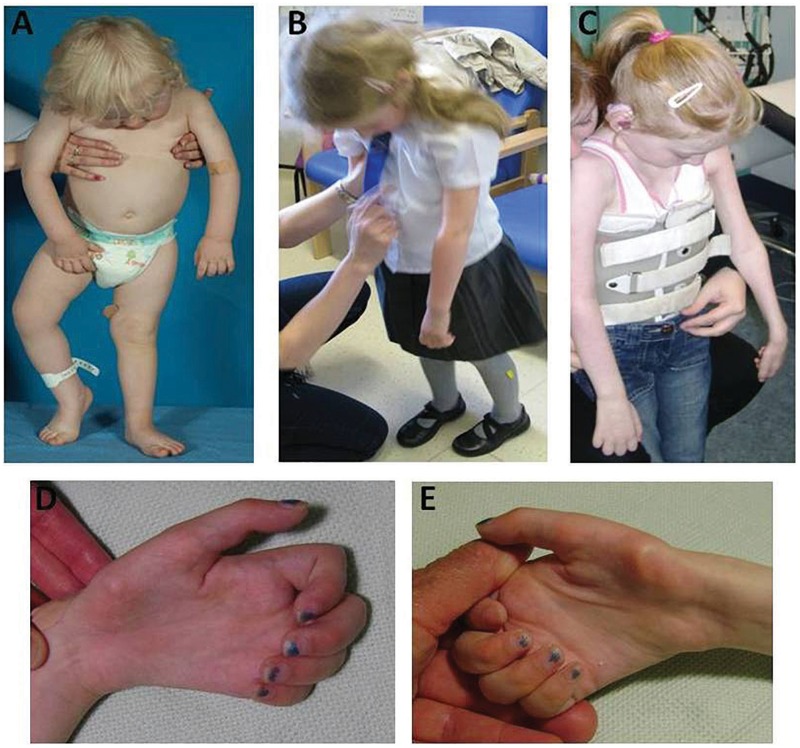
Phenotypic characteristics of Brown-Vialetto-Van Laere syndrome caused by mutations in *SLC52A2.* Severe weakness of neck extension and upper limbs with comparatively less weakness of lower limbs seen in Patient I1 at 1.8 years of age (**A**), Patient E4 at 5.8 years of age (**B**) and Patient E1 at 8.6 years of age (**C**). Symmetrical atrophy of intrinsic hand muscles of Patient E1’s left (**D**) and right (**E**) hands at 10.5 years of age.

Rapidly progressive upper limb weakness was noted after evidence of a sensory ataxic gait. An initial pattern of weakness of neck extension and the distal upper limbs progressed to involve the proximal upper limbs. A striking and consistent phenotype characterized by maintenance of the ability to walk (with head and trunk support) despite subgravity upper limb and neck strength was observed in all but one patient in this cohort, resulting from the comparatively milder lower limb weakness in contrast to the severe involvement of axial and upper limb muscles (Fig. 3).

Respiratory insufficiency developed in 13 patients (Table 1). Patient A3 developed subacute respiratory failure at 3.5 years of age and died despite ventilator support. Other clinical features consistently observed in this cohort include the absence of deep tendon reflexes and the presence of a flexor plantar response. Cognition was preserved in all 18 patients despite significant visual and hearing impairments.

### Biochemical studies

Plasma acylcarnitine profiles were performed in 17 of 18 patients with *SLC52A2* mutations before the initiation of high-dose oral riboflavin therapy. Ten of 17 patients tested (59%) had abnormal profiles (Supplementary material). A normalization of the acylcarnitine profile was documented after the initiation of riboflavin therapy in each of nine patients with abnormal profiles before riboflavin therapy (who had a repeat acylcarnitine profile performed) (Supplementary material), including within as short a time period as 2 weeks after the first riboflavin dose (Patient I1). Increases in riboflavin, flavin adenine dinucleotide and flavin mononucleotide levels (or increases in flavin adenine dinucleotide levels alone, when measured in isolation) were observed in 9 of 10 patients with measurements performed (in whole blood or plasma) before and after the initiation of riboflavin therapy (Supplementary material).

Respiratory chain studies were performed in muscle samples from five patients (Patients E1, E2, E5, E6 and A6) with abnormal results in two: Patient E2 with slightly decreased complex IV activity (0.012; reference range: 0.014–0.034) and Patient E6 with decreased complex I activity (0.089; reference range: 0.104–0.268).

### Neurophysiology

Neurophysiological studies were consistent with an axonal sensorimotor neuropathy in all 18 patients (Table 2). In patients with sequential nerve conduction studies, neurophysiological evidence of a sensory neuropathy clearly preceded that of a motor neuropathy (Table 2; numerical data in Supplementary material), and all patients demonstrated the same pattern of distribution of motor neuropathy with upper limbs more affected than lower limbs. This pattern is in contrast to inherited sensorimotor polyneuropathies that are typically length-dependent, with sensory symptoms and weakness in the lower limbs preceding and progressing to a greater degree than the upper limbs (Lindh *et al.*, 2005).

**Table 2:** Neurophysiological characteristics of patients with mutations in *SLC52A2*

								Family 1	
Patient	E1^a^	E2	E3	E4	E5	E6	E7	A1	A2
Age at nerve conduction testing	8 yrs	2 yrs	4 yrs	5 yrs	4 yrs	2 yrs	10 yrs	8 yrs	3 yrs
Sensory responses	Absent SNAPs in ULs and LLs	Low amplitude or absent SNAPs in ULs; absent in LLs	Low amplitude or absent SNAPs in ULs; low amplitude SNAPs in LLs	Low amplitude SNAPs in ULs; low amplitude or absent SNAPs in LLs	Normal	Absent SNAPs in ULs; low amplitude SNAPs in LLs	Low amplitude or absent SNAPs in ULs; absent in LLs	Absent SNAPs in ULs and LLs	Low amplitude SNAPs in ULs; absent in LLs
Motor responses	Low amplitude CMAPs in ULs and low amplitude or absent CMAPs in LLs	Low amplitude CMAPs in ULs and LLs	Absent CMAPs in ULs; normal in LLs	Low amplitude CMAPs in LLs (ULs not tested)	Low amplitude or absent CMAPs in ULs (LLs not tested)	Normal in LLs; (ULs not tested)	Low amplitude CMAPs in ULs; normal in LLs	Normal	Normal
Age at repeat nerve conduction testing	10 yrs	6 yrs			10 yrs	10 yrs		10 yrs	9 yrs
Sensory responses	Absent SNAPs in ULs and LLs	Absent SNAPs in ULs; (LLs not tested)			Low amplitude SNAPs in ULs; (LLs not tested)	Absent SNAPs in UL; (LL not tested)		Absent SNAPs in ULs and LLs	Absent SNAPs in ULs and LLs
Motor responses	Low amplitude CMAPs in ULs (LLs not tested)	Absent CMAPs in ULs; low amplitude CMAPs in LLs			Low amplitude CMAPs in ULs; (LLs not tested)	Low amplitude CMAPs in UL; (LL not tested)		Normal	Low amplitude CMAPs in ULs; normal in LLs

	Family 2		Family 3			Family 4			
Patient	A3	A4	A5^b^	A6^b^	A7	U1	U2	I1	L1
Age at nerve conduction testing	3 yrs	8 yrs	16 yrs	16 yrs	3 yrs	51 yrs	43 yrs	2 yrs	6 yrs
Sensory responses	Absent SNAPs in ULs and LLs	Low amplitude SNAPs in ULs; absent in LLs	Absent SNAPs in ULs and LLs	Absent SNAPs in ULs and LLs	Absent SNAPs in ULs and LLs	Low amplitude or absent SNAPs in UL; absent in LLs	Absent SNAPs in UL; (LL not tested)	Low amplitude SNAPs in ULs; absent in LLs	Low amplitude SNAPs in ULs and LLs
Motor responses		Normal	Low amplitude or absent CMAPs in ULs; normal in LLs	Low amplitude CMAPs in ULs; normal in LLs	Normal	Low amplitude CMAPs in ULs and LLs	Low amplitude or absent CMAPs in ULs; (LL not tested)	Low amplitude CMAPs in ULs and LLs	Normal
Age at repeat nerve conduction testing		16 yrs							
Sensory responses		Low amplitude SNAPs in ULs; absent in LLs							
Motor responses		Absent CMAPs in ULs; normal in LLs							

^a^Proband from Family D (Johnson *et al.*, 2012). ^b^Identical twins.

CMAP = compound motor action potential; LL = lower limb; SNAP = sensory nerve action potential; UL = upper limb; yr = year.

### Histopathology

**Figure 4:**
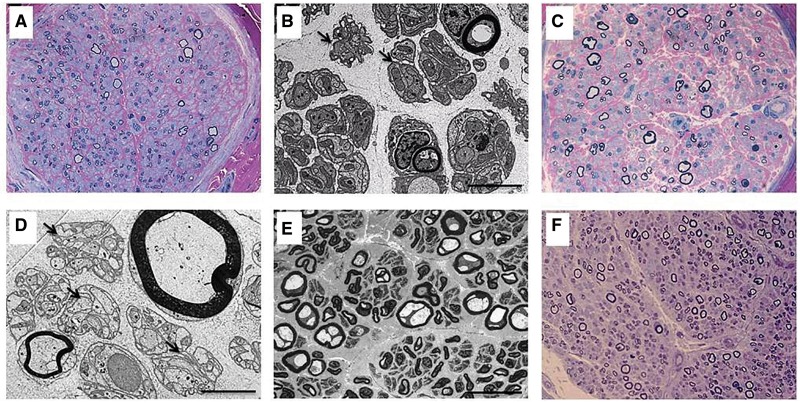
Sural nerve pathology in patients with mutations in *SLC52A2.* Resin semi-thin sections, stained with methylene blue–azure A and basic fuchsine; ×63 magnification (**A** and **C**) and toluidine blue; ×40 magnification (**F**) and electron microscopy examination (**B**, **D** and **E**) of sural nerve fascicle cross sections in Patient E2 at 2 years of age (**A** and **B**), Patient E3 at 4 years of age (**C** and **D**), Patient E5 at 4 years of age (**E**), and Patient A3 at 3 years of age (**F**) demonstrate a loss of myelinated axons, preferentially of large diameters (8–12 µm). The endoneurium is fibrotic, and there are no inflammatory infiltrates. Numerous redundant Schwannian profiles (Bands of Büngner) are discernible ultrastructurally (**B** and **D**, arrows) consistent with the loss of myelinated fibres. Several myelinated fibres in the biopsy of Patient E5 (**E**) appear vacuolated and slightly enlarged—probably an artefactual change not to be confused with giant axons. Note the striking absence of regeneration clusters in all biopsies. Scale bars: **B** and **D** = 5 µm; **E** = 50 µm.

Sural nerve biopsies were performed and adequately preserved in six patients (Patients E2, E3, E5, A3, A6 and I1), demonstrating findings consistent with a moderate to severe chronic axonal neuropathy, with accompanying fibrosis and variable ongoing degeneration (Fig. 4). Large myelinated fibres were consistently more severely affected. Regeneration was strikingly absent. Unmyelinated fibres were generally better preserved. There was no inflammation, pathological hypomyelination or demyelination, and barring artefacts, the surviving axons were morphologically normal or in a few instances atrophic (Supplementary material).

### Neuroimaging

Brain MRI was performed in 14 patients and revealed no structural or signal abnormalities. This finding is in contrast to reports of hyperintensity of brainstem nuclei (Koul *et al.*, 2006; Malheiros *et al.*, 2007), atrophy of the brainstem (Francis *et al.*, 1993; Koc *et al.*, 2003; Malheiros *et al.*, 2007) and atrophy of the cerebellum (Francis *et al.*, 1993; Koc *et al.*, 2003) in genetically undifferentiated cohorts of patients with Brown-Vialetto-Van Laere syndrome.

### Functional analyses of *SLC52A2* mutations

**Figure 5:**
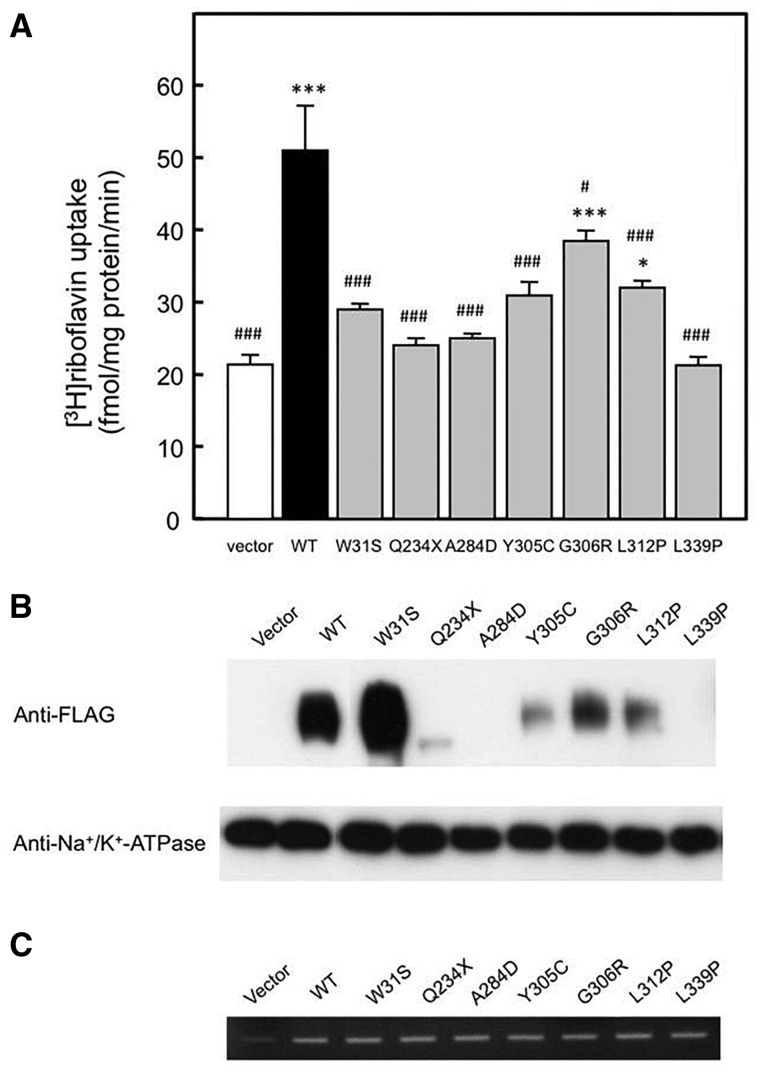
Functional studies of *SLC52A2* mutations. (**A**) Uptake of ^3^H-riboflavin by HEK293 cells transfected with empty vector (Vector), wild-type SLC52A2 (WT), SLC52A2 (92G > C; W31S), SLC52A2 (700C > T; Q234X), SLC52A2 (851C > A; A284D), SLC52A2 (914A > G; Y305C), SLC52A2 (916G > A; G306R), SLC52A2 (935T > C; L312P) and SLC52A2 (1016T > C; L339P). The cells were incubated with 5 nM ^3^H-riboflavin (pH 7.4) for 1 minute at 37°C. Each bar represents the mean ± SEM, *n* = 3. Data were analysed by Dunnett’s two-tailed test after one-way ANOVA. *P < 0.05, ***P < 0.001, significantly different from vector-transfected cells. ^#^*P < *0.05, ^###^*P < *0.001, significantly different from SLC52A2 (WT)-transfected cells. (**B**) Western blot analysis was performed using the crude membrane of HEK293 cells expressing empty vector, SLC52A2 (WT) and SLC52A2 variants. The crude membrane fractions were subjected to western blotting using antibodies against FLAG and Na^+^/K^+^-ATPase. Na^+^/K^+^-ATPase was used as an internal standard. (**C**) RNA expression of *SLC52A2* in HEK293 cells transfected with empty vector, *SLC52A2* (WT) and *SLC52A2* variants. Reverse transcription-PCR analysis was carried out using specific primer sets.

To assess the functional alterations caused by mutations in the *SLC52A2* gene, ^3^H-riboflavin transport activity was assessed using an *in vitro* transient expression system (Fig. 5A). ^3^H-Riboflavin uptake by the *SLC52A2* mutations p.W31S, p.Q234X, p.A284D, p.Y305C and p.L339P was completely abolished, and *SLC52A2* mutations p.G306R and p.L312P showed a moderate but significant decrease in ^3^H-riboflavin transport activity compared with wild-type *SLC52A2*. To determine whether the transport activity reduction was caused by the reduced expression of transporter proteins in the plasma membranes, western blot analysis was carried out using the crude membrane of HEK293 cells transiently transfected with these variants (Fig. 5B). The expression levels of *SLC52A2* mutants except for p.W31S were decreased compared with wild-type *SLC52A2*, which are well correlated with the reduction ratios of the transport activity for these variants. The dysfunctional p.W31S mutant was expressed in the plasma membrane. Moreover, to confirm the transfection efficiency of these cells, reverse transcription-PCR analysis was carried out. The RNA expressions of *SLC52A2* variants expressing cells were comparable to that of *SLC52A2* expressing cells (Fig. 5C). Native *SLC52A2* was only slightly observed in the cells transfected with empty vector.

### Response to riboflavin therapy

Sixteen patients have received high-dose riboflavin therapy ranging from 1 month to 20 months in duration, which has been well-tolerated and without evidence of toxicity. One patient died prior to being identified as harbouring *SLC52A2* mutations, and one patient refused riboflavin therapy. Fifteen patients have reported stable or improved function after the initiation of riboflavin therapy, and one patient was lost to follow-up (Supplementary material). Although most patients await repeat neurophysiological, pulmonary, visual evoked potential and audiometry evaluations while on high-dose riboflavin, we had the opportunity to study in detail two patients (Patients I1 and E1), whose significant and sustained clinical improvements are reported here.

Patient I1 presented at 22 months of age with a 6-month history of nystagmus, a 4-month history of an ataxic gait and a 3-week history of rapidly progressive bilateral hand and bulbar weakness. At the time of evaluation, he was unable to walk or hold a bottle. He then rapidly developed respiratory failure and was unable to swallow. Ophthalmological examination revealed bilateral optic atrophy, visual evoked response testing revealed vision loss and auditory brainstem response testing revealed bilateral sensorineural hearing loss. Elevations of C6, C8, C10 and C14:1 carnitine species were identified on plasma acylcarnitine profile testing (Supplementary material). Riboflavin was started within 8 days of initial presentation at a dose of 10 mg/kg/day and was increased to a dose of 50 mg/kg/day over the course of 4 weeks. Ten days after starting riboflavin, he was extubated and has remained stable without respiratory support. Within four weeks of starting riboflavin, he was feeding orally, holding his head upright, reaching for and grabbing toys and walking with trunk support. An acylcarnitine profile repeated 2 weeks after the initiation of riboflavin therapy was normal (Supplementary material).

Patient E1 was briefly described in relation to the identification of *SLC52A2* (Johnson *et al.*, 2012). She presented with sensory ataxia at 18 months of age and then developed rapidly progressive upper limb weakness, hearing loss, vision loss and respiratory insufficiency at age 6 years. She was found to have mutations in *SLC52A2* at age 10 years and was started on a riboflavin dose of 10 mg/kg/day that was titrated up to a dose of 50 mg/kg/day over the course of 12 weeks. Biochemical and clinical improvements observed after 3 months of riboflavin therapy include a normalization of the acylcarnitine profile (Supplementary material), a clear improvement in audiometry testing (responsive to 40–55 dB at 8 kHz after 3 months of riboflavin therapy, compared with 80 dB at 8 kHz prior to riboflavin therapy) and mild improvements in pulmonary function and visual evoked potentials (Supplementary material). This is in contrast with previous sequential audiometry testing, pulmonary function testing and visual evoked potentials testing that had documented continual functional decline. There were notable improvements in growth of weight, height, hair and shoe size, which had remained unchanged between 6 and 10 years of age. After 20 months of riboflavin therapy, pulmonary function and audiometry have remained stably improved. Motor function improvements have been more marked and include the ability to sit and stand independently for the first time in 3 years.

## Discussion

Dr. Charles Brown prefaced his presentation of a case of ‘infantile amyotrophic lateral sclerosis of the family type’ at the meeting of the American Neurological Association in 1894 by stating that the ‘case opens up a new type of cases for study and that it is a sign post not to be overlooked’ (Brown, 1894). We have described 18 patients with mutations in the riboflavin transporter gene *SLC52A2* who demonstrate a striking clinical phenotype of sensory ataxia and upper limb, axial and respiratory weakness as a result of an axonal sensorimotor peripheral neuropathy; and a cranial neuropathy affecting cranial nerves II (optic atrophy), VIII (hearing loss) and XII (tongue fasciculations ± tongue weakness and atrophy). We also report the consistent neurophysiological profile and sural nerve pathology associated with mutations in RFVT2 and demonstrate that *SLC52A2* mutations cause reduced riboflavin uptake and reduced riboflavin transporter protein expression. As three patients with mutations in *SLC52A2* were identified from an undiagnosed cohort of 63 patients with cranial neuropathies and sensorimotor neuropathy ± respiratory insufficiency which was Sanger sequenced for *SLC52A1*, *SLC52A2* and *SLC52A3*, and none of the patients in this cohort were found to harbour mutations in *SLC52A1* or *SLC52A3*, it seems that *SLC52A2* is perhaps the most common cause of Brown-Vialetto-Van Laere syndrome. It is also notable that among eight individuals who had been separately identified as not only having cranial neuropathies and sensorimotor neuropathy ± respiratory insufficiency but also clinical features evocative of the initial report of *SLC52A2* (Johnson *et al.*, 2012)—including sensory ataxia, predominantly upper limb and axial weakness, hearing loss and optic atrophy—all eight were found to harbour mutations in *SLC52A2* (and no mutations in *SLC52A1* or *SLC52A3*), suggesting that mutations in this riboflavin transporter gene may be quite specific to the distinct phenotype presented here.

**Figure 6:**
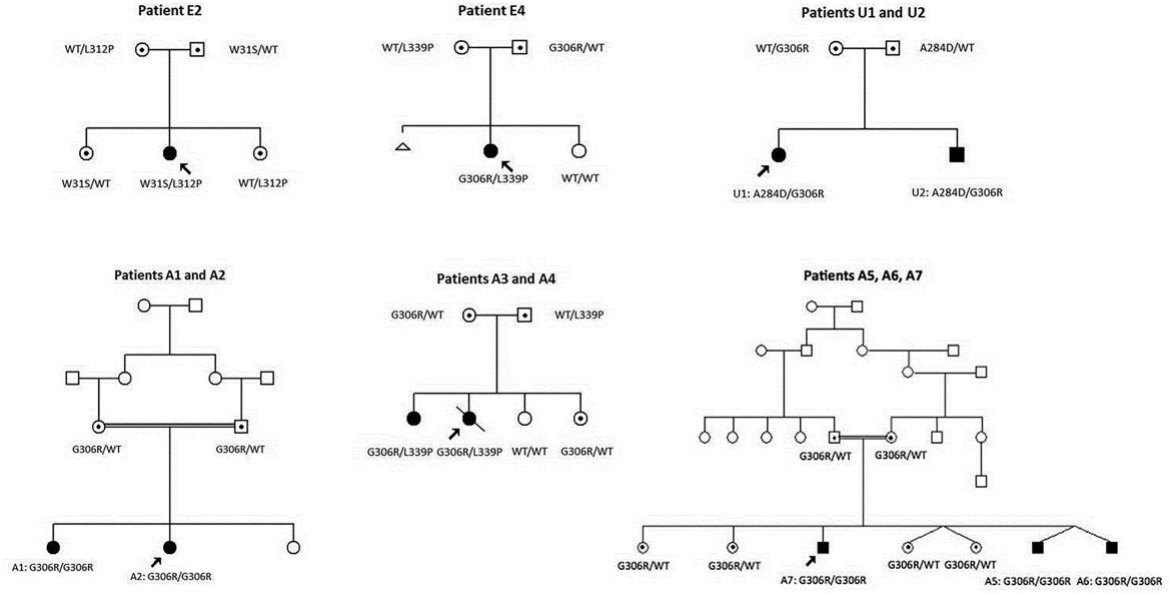
Autosomal recessive inheritance of *SLC52A2* mutations. Pedigrees and corresponding *SLC52A2* mutations for Patients E2, E4, U1, U2, A1, A2, A3, A4, A5, A6 and A7. Squares denote males, circles females, shaded shapes affected individuals, and shapes with dots carriers. Double bars indicate consanguineous unions, and arrows indicate probands.

Brown-Vialetto-Van Laere syndrome associated with *SLC52A2* mutations is inherited as an autosomal recessive condition (Fig. 6), which helps to differentiate it from other optico-acoustic neuropathies including autosomal dominantly inherited *OPA1* or *MFN2* mutations, X-linked *PRPS1* mutations and mitochondrially inherited neuropathy caused by mitochondrial DNA mutations (such as Leber’s hereditary optic neuropathy or the syndrome of neuropathy, ataxia and retinitis pigmentosa known as ‘NARP’). Brown-Vialetto-Van Laere syndrome resulting from mutations in *SLC52A2* can also be clearly distinguished from other neuropathies that may present with predominantly upper limb weakness, including distal hereditary motor neuropathies caused by mutations in *BSCL2* or *GARS*, given that autosomal recessive mode of inheritance, the presence of a sensory neuropathy (in combination with a motor neuropathy), optic atrophy, hearing loss and respiratory insufficiency seen in our *SLC52A2*-specific cohort are not present in neuropathies caused by mutations in *BSCL2* or *GARS* (Rossor *et al.*, 2012).

Recessive mutations in the *SLC52A3* gene can also result in Brown-Vialetto-Van Laere syndrome; however, there are differences in the phenotype of patients harbouring *SLC52A2* mutations compared with the phenotype of patients with *SLC52A3* mutations. Early onset weakness in the upper limbs and neck is almost invariably seen in patients with mutations in *SLC52A2*, in contrast to those patients with *SLC52A3* mutations or genetically unclassified Brown-Vialetto-Van Laere syndrome, in whom the onset of weakness is often more generalized (Green *et al.*, 2010; Bosch *et al.*, 2011). Another distinctive feature of patients with *SLC52A2* mutations is a lack of upper motor neuron signs in the lower limbs, a commonly reported clinical feature of Brown-Vialetto-Van Laere syndrome (Gallai *et al.*, 1981; Hawkins *et al.*, 1990; Francis *et al.*, 1993; Voudris *et al.*, 2002; De Grandis *et al.*, 2005; Dipti *et al.*, 2005; Koul *et al.*, 2006) and, in particular, reported in patients with mutations in *SLC52A3* (Green *et al.*, 2010). Furthermore, the most common initial presenting symptom reported in case reports and reviews of Brown-Vialetto-Van Laere syndrome is bilateral hearing loss (Gallai *et al.*, 1981; Sathasivam, 2008). In contrast, the most common presenting symptom in our cohort of patients with *SLC52A2* mutations is an ataxic gait, which in young children is likely the first indication of an underlying sensory neuropathy. Some patients presented with nystagmus, likely related to optic atrophy and serving as a heralding symptom before a precipitous decline in upper limb strength, respiratory function and hearing. Given these findings, it is imperative that neurologists evaluating children with clinical evidence of sensory ataxia and/or nystagmus/optic atrophy consider the possibility of an underlying riboflavin transporter defect.

It is notable that normal nerve conduction velocities have been reported in patients diagnosed with Brown-Vialetto-Van Laere syndrome (Voudris *et al.*, 2002; Dipti *et al.*, 2005; Koul *et al.*, 2006) and that sensory loss and abnormalities on sensory nerve conduction studies have not been commonly recognized as part of the phenotype of Brown-Vialetto-Van Laere syndrome in cohorts of genetically undifferentiated patients (Sathasivam, 2008). This is in stark contrast with our cohort of patients with mutations in *SLC52A2* who presented with sensory ataxia and nerve conduction studies revealing an axonal sensorimotor neuropathy. Going forward, it seems advisable to adopt a new nomenclature for accurately studying the phenotypic subgroups falling under the term ‘Brown-Vialetto-Van Laere’ syndrome. To this end, the terms ‘riboflavin transporter deficiency, type 1 (hRFT1), 2 (hRFT2) and 3 (hRFT3)’ were recommended (Bosch *et al.*, 2012). Using the new protein nomenclature and aiming to achieve improved clarity, we recommend that the term ‘riboflavin transporter deficiency, type 2’ be used to correspond to the *SLC52A2* encoded RFVT2 (formerly RFT3) and ‘riboflavin transporter deficiency, type 3’ to correspond to the *SLC52A3* encoded RFVT3 (formerly RFT2).

The identification of mutations in riboflavin transporter genes in this subset of patients with Brown-Vialetto-Van Laere syndrome has uncovered a pathophysiological mechanism for this neurodegenerative condition, making possible a therapeutic intervention for patients for whom no disease modifying therapy had been available previously (Sathasivam, 2008). Riboflavin (vitamin B_2_) is a precursor of flavin mononucleotide and flavin adenine dinucleotide, both cofactors important for carbohydrate, amino acid and lipid metabolism (Gropper, 2012). Flavin adenine dinucleotide is an electron acceptor in acyl-dehydrogenation reactions for mitochondrial fatty acid beta-oxidation and branched chain amino acid catabolism (Gregersen *et al.*, 2008), and both flavin adenine dinucleotide and flavin mononucleotide are required for normal respiratory chain function. The riboflavin transporter RFVT3 is reported to be a saturable, energy-dependent carrier (Moriyama, 2011). Preliminary human tissue studies of the *SLC52A2* encoded RFVT2 demonstrate a relatively higher expression in the brain and spinal cord than in the small intestine (Yao *et al.*, 2010).

The sural nerve biopsies of six patients from this *SLC52A2*-specific cohort demonstrate a preferential loss of large diameter myelinated axons, thus providing a neuropathological correlate to the clinical finding of absent deep tendon reflexes and the highly prevalent symptom of sensory ataxia observed in these patients. Furthermore, the distinct lack of regeneration in these biopsies points toward a potential underlying neuronopathy and, in particular, may indicate involvement of the dorsal root ganglion cells. As this is the first report of sural nerve pathology in patients with mutations in RFVT2, it remains to be seen if these findings are indeed *SLC52A2*-specific. Previous pathological descriptions in patients with Brown-Vialetto-Van Laere syndrome are limited to rare post-mortem studies of genetically undifferentiated cohorts and include findings of neuronal loss and degeneration in lower cranial nerve nuclei (VII–XII), depletion of anterior horn cells and degeneration of spinocerebellar and pyramidal tracts (Brucher *et al.*, 1981). A severe depletion of motor root axons, an absence of large motor neurons and an almost complete loss of fibres in Clarke’s column and the posterior horns of the spinal column have also been reported (Francis *et al.*, 1993), including evidence of these findings in the cervical and upper thoracic levels of the spinal cord with marked sparing of the lumbosacral levels (Rosemberg *et al.*, 1982).

Further studies may provide information for effectively optimizing riboflavin transport through RFVT1 and RFVT3 [known riboflavin transporters with high expression in the small intestine (Yao *et al.*, 2010) that presumably remain functional in patients with *SLC52A2* mutations] as well as riboflavin uptake through diffusion, due to the potential of saturation of these transporters. Given that the goal of high-dose oral riboflavin therapy is optimal recovery of axonal damage, which may be secondary to an underlying neuronopathy (affecting dorsal root ganglia and anterior horn neurons) or a primary axonopathy (resulting in the sensorimotor neuropathy, sensorineural hearing loss and optic atrophy seen in this condition), these are important research questions to answer.

Here we have reported significant clinical and biochemical improvements observed after the initiation of high-dose oral riboflavin therapy in patients with mutations in the riboflavin transporter gene *SLC52A2* including the apparently life-saving clinical improvement evident within 4 weeks of high-dose riboflavin therapy in a 23-month-old infant (Patient I1). The degree of improvement observed in this young patient may be a function of the short time period between the onset of his symptoms and the initiation of high-dose riboflavin. Given these findings, we suggest that siblings of affected individuals be promptly screened for mutations in *SLC52A2*, particularly as the identification of presymptomatic individuals would provide a therapeutic opportunity and could determine if presymptomatic high-dose riboflavin therapy could decrease symptoms of this axonal sensorimotor neuropathy or potentially prevent their emergence. Although the degree of clinical improvement possible in older affected individuals—with riboflavin therapy initiated well after the onset of symptoms—remains to be seen, the clinical and biochemical improvements observed with high-dose oral riboflavin therapy in our *SLC52A2*-specific cohort, as well as previously described patients with mutations in *SLC52A3* (Anand *et al.*, 2012; Bosch *et al.*, 2012; Ciccolella *et al.*, 2012), are noteworthy. Taken together, and considering the low risk of toxicity of oral riboflavin even at high doses (Alhadeff *et al.*, 1984) (as the excess is readily excreted in the urine), we suggest that patients presenting with the clinical phenotype associated with mutations in *SLC52A2* described here, as well as phenotypes described in association with *SLC52A3*, have high-dose oral riboflavin therapy initiated early. It is the timely consideration of a diagnosis of a riboflavin transporter deficiency and the rapid initiation of high-dose riboflavin therapy, even while sequencing of riboflavin transporter genes is in progress, that may hold the greatest promise of preventing and potentially reversing the progression of this hitherto elusive and relentlessly progressive neurodegenerative condition.

## Supplementary Material

Supplementary Data
