# Supplementary material for: Treatable childhood neuronopathy caused by mutations in riboflavin transporter RFVT2
Source: Brain. 2013 Nov 15;137(1):44–56. doi: 10.1093/brain/awt315 (PMC3891447; doi:10.1093/brain/awt315)
Supplement: Supplementary Data [file supp_137_1_44__index.html]

Treatable childhood neuronopathy caused by mutations in riboflavin transporter RFVT2 — Supplementary Data 

# Treatable childhood neuronopathy caused by mutations in riboflavin transporter RFVT2

## Supplementary Data

files

**Files in this Data Supplement:**

- Supplementary Data - docx file
