# Supplementary material for: Treatable childhood neuronopathy caused by mutations in riboflavin transporter RFVT2
Source: Brain. 2013 Nov 15;137(1):44–56. doi: 10.1093/brain/awt315 (PMC3891447; doi:10.1093/brain/awt315)
Supplement: Supplementary Data [file supp_awt315_Supplementary_Material.docx]

**Supplementary Material**

Table of Contents:

Supplementary Table 1: Biochemical profiles prior to riboflavin therapy...................................2-3

Supplementary Table 2: Biochemical profiles following the initiation of riboflavin therapy….4-5

Supplementary Table 3: Observed responses to riboflavin therapy………….............................6-7

Supplementary Table 4: Response to riboflavin therapy in Patient E1………………..………….8

Supplementary Table 5: Sural nerve pathology findings in patients with mutations in *SLC52A2*..9

Supplementary Figure 1: Scatterplot of g ratio measurements as a function of axon diameter....10

Supplementary Table 6: Numerical data for patients with repeat neurophysiology studies…..11

Supplementary Table 7: Primer sequences used in Sanger sequencing *SLC52A1*, *SLC52A2* and *SLC52A3*........................................................................................................................................12

Sequence Traces.......................................................................................................................13-20

Exome Sequencing Methods....................................................................................................21-24

Functional Analyses of *SLC52A2* Mutations - Materials and Methods……..………….........24-25

Supplementary Table 8: Primers used for site-directed mutagenesis………...………………….26

References......................................................................................................................................27

| **Supplementary Table 1 Biochemical profiles prior to riboflavin therapy** |  |  |  |  |  |  |  | Family 1 | |  |  |  |  |  |  |  | |
| --- | --- | --- | --- | --- | --- | --- | --- | --- | --- | --- | --- | --- | --- | --- | --- | --- | --- |
| **Patient** | **E1**^a^ | **E2** | **E3** | **E4** | **E5**** | **E6** | **E7** | **A1** | **A2** |  |  |  |  |  |  |  | |
| **Age at time of biochemical testing** | 10 yrs | 6 yrs | 5 yrs | 5 yrs | 3.5 | 17 yrs | 22 yrs | 10 yrs | 9 yrs |  |  |  |  |  |  |  | |
| **Riboflavin [reference range]** | 7* | 8.5* | 14.9* | 2.9* | nd | nd | 7.2* | nd | nd |  |  |  |  |  |  |  | |
| **FAD [reference range]** | 201 [174-471] | 185 [174-471] | 87.2* | 253 [174-471] | nd | nd | 311 [174-471] | 267 [174-471] | 253 [174-471] |  |  |  |  |  |  |  | |
| **FMN [reference range]** | <4* | 7.4* | 4.5* | 5.8* | nd | nd | 9.9* | nd | nd |  |  |  |  |  |  |  | |
| **C0-carnitine [reference range]** | 18.3 [<52.4] | nml | nml | **14** [17-55] | nml | 41 [23-75] | 37.0 [<52.4] | 16.8 [13-56] | 25.6 [13-56] |  |  |  |  |  |  |  | |
| **C2-carnitine [reference range]** | **19.3** [<11.5] | nml | nml | 10 [10-27.8] | nml | 20.8 [10-34.5] | **14.7** [<11.5] | 4.1 [2.8-22.5] | 3.7 [2.8-22.5] |  |  |  |  |  |  |  | |
| **C4-carnitine [reference range]** | **1.72** [<0.60] | nml | nml | **0.5** [0.11-0.49] | nml | 1.69 [0.43-2.44] | 0.44 [<0.60] | 0.35 [0.12-0.67] | 0.24 [0.12-0.67] |  |  |  |  |  |  |  | |
| **C5-carnitine [reference range]** | **0.34** [<0.24] | nml | nml | **0.56** [0.08-0.35] | nml | 0.21 [0.1-0.53] | 0.06 [<0.24] | **0.37** [<0.28] | **3.82** [<0.28] |  |  |  |  |  |  |  | |
| **C6-carnitine [reference range]** | **0.79** [<0.12] | nml | nml | **0.19** [0.02-0.11] | nml | 0.23 [0.06-0.31] | 0.06 [<0.12] | **0.58** [<0.13] | **0.31** [<0.13] |  |  |  |  |  |  |  | |
| **C8-carnitine [reference range]** | **1.64** [<0.24] | nml | nml | **0.49** [0-0.18] | nml | 0.00 [0.04-0.2] | 0.17 [<0.24] | **1.31** [<0.24] | **0.29** [<0.24] |  |  |  |  |  |  |  | |
| **C10:1-carnitine [reference range]** | 0.47 [<0.51] | nml | nml | nd | nml | nd | 0.16 [<0.51] | **0.79** [<0.5] | **0.68** [<0.5] |  |  |  |  |  |  |  | |
| **C10-carnitine [reference range]** | **2.15** [<0.40] | nml | nml | **0.42** [<0.2] | nml | nd | 0.36 [<0.40] | **1.39** [<0.4] | **0.61** [<0.4] |  |  |  |  |  |  |  | |
| **C12-carnitine [reference range]** | **1.64** [<0.22] | nml | nml | nd | nml | nd | 0.15 [<0.22] | 0.12 [<0.62] | **0.79** [<0.62] |  |  |  |  |  |  |  | |
| **C14:1-carnitine [reference range]** | **2.19** [<0.25] | nml | nml | nd | nml | nd | **0.33** [<0.25] | 0.16 [<0.73] | 0.11 [<0.73] |  |  |  |  |  |  |  | |
| **C14-carnitine [reference range]** | **0.94** [<0.12] | nml | nml | 0.14 [0.09-0.25] | nml | 0.12 [0.02-0.21] | 0.05 [<0.12] | 0.03 [<0.34] | 0.12 [<0.34] |  |  |  |  |  |  |  | |
| **C16-carnitine [reference range]** | **0.61** [<0.28] | nml | nml | 0.8 [0.4-1.7] | nml | 0.8 [0.5-2.2] | 0.09 [<0.28] | 0.07 [<0.64] | 0.08 [<0.64] |  |  |  |  |  |  |  | |
| **C18:1-carnitine [reference range]** | **1.45** [<0.40] | nml | nml | nd | nml | nd | 0.15 [<0.40] | nd | nd |  |  |  |  |  |  |  |  |
| **C18:2-carnitine [reference range]** | **0.44** [<0.23] | nml | nml | nd | nml | nd | 0.04 [<0.23] | nd | nd |  |  |  |  |  |  |  |  |

|  | Family 2 | | Family 3 | | | Family 4 | |  |  |
| --- | --- | --- | --- | --- | --- | --- | --- | --- | --- |
| **Patient** | **A3** | **A4** | **A5**^b^ | **A6**^b^ | **A7** | **U1** | **U2** | **I1** | **L1** |
| **Age at time of biochemical testing** | 3.5 yrs | 15 yrs | 16 yrs | 16 yrs | 21 yrs | 52 yrs | 44 yrs | 1.8 yrs | na |
| **Riboflavin [reference range]** | nd | nd | nd | nd | nd | nd | nd | 9.8 [3.9-49.0] | nd |
| **FAD [reference range]** | nd | 245 [174-471] | 369 [174-471] | 310 [174-471] | 337 [174-471] | 256 [137-370] | 242 [137-370] | 64.4 [46.0-114.0] | nd |
| **FMN [reference range]** | nd | nd | nd | nd | nd | nd | nd | **2.5** [2.8-21.4] | nd |
| **C0-carnitine [reference range]** | 38.8 [13-56] | 30.2 [13-56] | 21.9 [13-56] | 18.3 [13-56] | 27.3 [13-56] | nd | nd | 24.30 [22.30-54.80] | nd |
| **C2-carnitine [reference range]** | 9.5 [2.8-22.5] | 11.1 [2.8-22.5] | 3.1 [2.8-22.5] | 3.4 [2.8-22.5] | 5.1 [2.8-22.5] | 8.69 [3.74-16.56] | 5.70 [3.74-16.56] | 4.97 [3.40-13.00] | nd |
| **C4-carnitine [reference range]** | 0.65 [0.12-0.67] | 0.6 [0.12-0.67] | 0.1 [0.12-0.67] | 0.03 [0.12-0.67] | 0.28 [0.12-0.67] | 0.29 [0.00-0.45] | **0.46** [0.00-0.45] | 0.25 [0.07-0.58] | nd |
| **C5-carnitine [reference range]** | 0 [<0.28] | **0.31** [<0.28] | 0.1 [<0.28] | 0.08 [<0.28] | 0.26 [<0.28] | 0.15 [0.00-0.30] | **0.96** [0.00-0.30] | 0.13 [0.04-0.22] | nd |
| **C6-carnitine [reference range]** | 0.12 [<0.13] | **0.27** [<0.13] | 0.04 [<0.13] | 0.04 [<0.13] | 0.07 [<0.13] | **0.21** [0.00-0.12] | 0.09 [0.00-0.12] | **0.14** [0.02-0.12] | nd |
| **C8-carnitine [reference range]** | **0.34** [<0.24] | **0.7** [<0.24] | 0.09 [<0.24] | 0.11 [<0.24] | 0.16 [<0.24] | **0.40** [0.00-0.23] | **0.24** [0.00-0.23] | **0.25** [0.04-0.22] | nd |
| **C10:1-carnitine [reference range]** | 0.19 [<0.5] | **0.53** [<0.5] | 0.1 [<0.5] | 0.13 [<0.5] | 0.08 [<0.5] | **0.40** [0.00-0.31] | 0.25 [0.00-0.31] | 0.15 [0.04-0.22] | nd |
| **C10-carnitine [reference range]** | **0.52** [<0.4] | **0.8** [<0.4] | 0.13 [<0.4] | 0.16 [<0.4] | 0.2 [<0.4] | **0.45** [0.00-0.31] | 0.29 [0.00-0.31] | **0.36** [0.04-0.30] | nd |
| **C12-carnitine [reference range]** | 0.16 [<0.62] | 0.09 [<0.62] | 0.01 [<0.62] | 0.03 [<0.62] | 0.02 [<0.62] | 0.09 [0.00-0.12] | 0.06 [0.00-0.12] | 0.11 [0.04-0.14] | nd |
| **C14:1-carnitine [reference range]** | 0.16 [<0.73] | 0.18 [<0.73] | 0.06 [<0.73] | 0.09 [<0.73] | 0.05 [<0.73] | **0.17** [0.00-0.16] | 0.07 [0.00-0.16] | **0.20** [0.02-0.18] | nd |
| **C14-carnitine [reference range]** | 0.06 [<0.34] | 0 [<0.34] | 0 [<0.34] | 0.04 [<0.34] | 0.01 [<0.34] | **0.06** [0.00-0.05] | 0.03 [0.00-0.05] | 0.06 [0.00-0.08] | nd |
| **C16-carnitine [reference range]** | 0.03 [<0.64] | 0.11 [<0.64] | 0.04 [<0.64] | 0.09 [<0.64] | 0.05 [<0.64] | 0.09 [0.00-0.10] | 0.08 [0.00-0.10] | 0.11 [0.06-0.24] | nd |
| **C18:1-carnitine [reference range]** | nd | nd | nd | nd | nd | 0.12 [0.00-0.17] | 0.08 [0.00-0.17] | 0.22 [0.06-0.28] | nd |
| **C18:2-carnitine [reference range]** | nd | nd | nd | nd | nd | 0.08 [0.00-0.10] | 0.06 [0.00-0.10] | 0.08 [0.02-0.18] | nd |

^a^proband from Family D (Johnson *et al.*, 2012); ^b^identical twins; *no reference range (at laboratory where testing performed); **refused riboflavin therapy

FAD = flavin adenine dinucleotide; FMN = flavin mononucleotide; na = not applicable; nd = not done; nml = normal (actual values not available); yrs = years

Concentrations of carnitine species are in micromolarity per litre. Concentrations of riboflavin, FAD and FMN are in nanomoles per litre. Abnormal results in bold. Reference ranges for respective laboratories in brackets.

| **Supplementary Table 2 Biochemical profiles following the initiation of riboflavin therapy** |  |  |  |  |  |  |  | Family 1 | |
| --- | --- | --- | --- | --- | --- | --- | --- | --- | --- |
| **Patient** | **E1**^a^ | **E2** | **E3** | **E4** | **E5**** | **E6** | **E7** | **A1** | **A2** |
| **Riboflavin [reference range]** | 71.9* | nd | 28.4 [3.9-49.0] | 24.3* | na | nd | 14.7* | nd | nd |
| **FAD [reference range]** | 313 [174-471] | nd | **126.6** [46.0-114.0] | 367 [174-471] | na | nd | 359 [174-471] | 407 [174-471] | 383 [174-471] |
| **FMN [reference range]** | 68.6* | nd | 5.6 [2.8-21.4] | 73.5* | na | nd | 31.5* | nd | nd |
| **C0-carnitine [reference range]** | 22 [17-55] | nd | nd | 37 [17.0-55.0] | na | nd | 50 [<52.4] | 9 [13-56] | 17 [13-56] |
| **C2-carnitine [reference range]** | 16.7 [10-27.8] | nd | nd | 19.7 [10-27.8] | na | nd | 5.2 [<11.5] | 2 [2.8-22.5] | 3 [2.8-22.5] |
| **C4-carnitine [reference range]** | 0.32 [0.11-0.49] | nd | nd | 0.22 [0.11-0.49] | na | nd | 0.39 [<0.60] | nd | nd |
| **C5-carnitine [reference range]** | 0.15 [0.08-0.35] | nd | nd | 0.17 [0.08-0.35] | na | nd | 0.05 [<0.24] | nd | nd |
| **C6-carnitine [reference range]** | 0.03 [0.02-0.11] | nd | nd | 0.04 [0.02-0.11] | na | nd | 0.03 [<0.12] | <0.1 [<0.13] | <0.1 [<0.13] |
| **C8-carnitine [reference range]** | 0.14 [0-0.18] | nd | nd | 0.16 [0-0.18] | na | nd | 0.06 [<0.24] | <0.1 [<0.24] | <0.1 [<0.24] |
| **C10:1-carnitine [reference range]** | nd | nd | nd | nd | na | nd | 0.05 [<0.51] | <0.1 [<0.5] | <0.1 [<0.5] |
| **C10-carnitine [reference range]** | nd | nd | nd | nd | na | nd | 0.09 [<0.40] | 0.1 [<0.4] | 0.1 [<0.4] |

|  | Family 2 | | Family 3 | | | Family 4 | |  |  |
| --- | --- | --- | --- | --- | --- | --- | --- | --- | --- |
| **Patient** | **A3^b^** | **A4** | **A5**^c^ | **A6**^c^ | **A7** | **U1** | **U2** | **I1** | **L1** |
| **Riboflavin [reference range]** | nd | nd | nd | nd | nd | nd | **219.8** [6.2-39.0] | nd | nd |
| **FAD [reference range]** | nd | 324 [174-471] | 396 [174-471] | 380 [174-471] | 337 [174-471] | nd | nd | nd | nd |
| **FMN [reference range]** | nd | nd | nd | nd | nd | nd | nd | nd | nd |
| **C0-carnitine [reference range]** | nd | 27 [13-56] | 21 [13-56] | 20 [13-56] | 27 | nd | nd | nml | nd |
| **C2-carnitine [reference range]** | nd | 5 [2.8-22.5] | 4 [2.8-22.5] | 5 [2.8-22.5] | 5 | 7.30 [4.04-12.19] | 4.64 [4.04-12.19] | nd | nd |
| **C4-carnitine [reference range]** | nd | nd | nd | nd | nd | 0.07 [<0.38] | 0.19 [<0.38] | nd | nd |
| **C5-carnitine [reference range]** | nd | nd | nd | nd | nd | 0.04 [<0.30] | **0.35 [<0.30**] | nml | nd |
| **C6-carnitine [reference range]** | nd | 0.1 [<0.13] | <0.1 [<0.13] | 0 [<0.13] | 0.1 [<0.13] | 0.02 [<0.09] | 0.02 [<0.09] | nml | nd |
| **C8-carnitine [reference range]** | nd | 0.1 [<0.24] | <0.1 [<0.24] | 0.1 [<0.24] | 0.2 [<0.24] | 0.12 [<0.65] | 0.04 [<0.65] | nml | nd |
| **C10:1-carnitine [reference range]** | nd | 0.1 [<0.5] | <0.1 [<0.5] | 0.1 [<0.5] | 0.1 [<0.5] | 0.11 [<0.81] | 0.07 [<0.81] | nd | nd |
| **C10-carnitine [reference range]** | nd | 0.2 [<0.4] | <0.1 [<0.4] | 0.1 [<0.4] | 0.2 [<0.4] | 0.17 [<0.51] | 0.05 [<0.51] | nd | nd |

^a^proband from Family D (Johnson *et al.*, 2012); ^b^died before starting riboflavin; ^c^identical twins; *no reference range (at laboratory where testing performed); **refused riboflavin therapy

FAD = flavin adenine dinucleotide; FMN = flavin mononucleotide; na = not applicable; nd = not done; nml = normal (actual values not available)

Concentrations of carnitine species are in micromolarity per litre. Concentrations of riboflavin, FAD and FMN are in nanomoles per litre. Abnormal results in bold. Reference ranges for respective laboratories in brackets.

| **Supplementary Table 3 Observed responses to riboflavin therapy** |  |  |  |  |  |  | Family 1 | |
| --- | --- | --- | --- | --- | --- | --- | --- | --- |
| **Patient** | **E1^a^** | **E2** | **E3** | **E4** | **E6*** | **E7** | **A1** | **A2** |
| **Age at first symptom** | 1.5 yr | 1 yr | 3.5 yrs | 1.5 yrs | 0.6 yrs | 2 yrs | 8 yrs | 3 yrs |
| **Age at initiation of high-dose riboflavin therapy** | 10.6 yrs | 6 yrs | 5.4 yrs | 5 yrs | 17.5 yrs | 22.4 yrs | 10 yrs | 9 yrs |
| **Present dose of riboflavin** | 50 mg/kg/day | 10 mg/kg/day | 7 mg/kg/day | 40 mg/kg/day | 300 mg/day | 1,500 mg/day | 23 mg/kg/day | 26 mg/kg/day |
| **Length of time at present dose** | 12 months | 15 months | 15 months | 11 months | 13 months | 2 weeks | 7 months | 7 months |
| **Observed response(s)** | able to go from supine to sitting and standing independently (skills lost at age 9 years); stronger/louder voice; *further details in Supplementary Table 4* | *lost to follow-up* | stable functioning; no changes noted | can walk independently; can jump (skill lost at age 4 years); hearing grossly better | stable functioning; no changes noted | increased sensation in legs | normalisation of both pure tone audiometry; normalisation of brainstem auditory evoked responses; fewer falls | stable functioning; decreased fatigability reported |

|  |  | Family 3 | | | Family 4 | |  |  |
| --- | --- | --- | --- | --- | --- | --- | --- | --- |
| **Patient** | **A4** | **A5^b^** | **A6^b^** | **A7** | **U1** | **U2** | **I1** | **L1** |
| **Age at first symptom** | 5 yrs | 3 yrs | 3 yrs | 5 yrs | 2 yrs | 4 yrs | 1.3 yrs | 3 yrs |
| **Age at initiation of high-dose riboflavin therapy** | 15 yrs | 16 yrs | 16yrs | 21 yrs | 52 yrs | 44 yrs | 1.8 yrs | 6.5 yrs |
| **Present dose of riboflavin** | 21 mg/kg/day | 1,000 mg/day | 1,000 mg/day | 1,000 mg/day | 400 mg/day** | 800 mg/day** | 60 mg/kg/day*** | 10 mg/kg/day |
| **Length of time at present dose** | 7 months | 7 months | 7 months | 7 months | 6 months | 6 months | 2 months*** | 13 months |
| **Observed response(s)** | stable functioning; decreased fatigability reported | better at transferring; stronger kick; significant decrease in ventilator pressures | stable functioning; no changes noted | stable functioning; no changes noted | stable functioning; no changes noted | stable functioning; no changes noted | improved upper limb and neck strength: antigravity shoulder abduction, elbow flexion and extension and neck extension; can walk with truncal support; stronger/louder voice; decreased nystagmus; *further details in main text* | less frequent falls |

kg = kilograms; mg = milligrams; yr = year; yrs = years

^a^proband from Family D (Johnson *et al.*, 2012); ^b^identical twins

*had been started on low-dose riboflavin (3 mg/kg/day) at age 15 years by patient’s metabolic consultant (SR) for evidence of a mitochondrial disorder. [Riboflavin dose increased to 300 mg/day (10 mg/kg/day) when *SLC52A2* mutations found.]

**unable to tolerate further increase in dose due to reported associated gastrointestinal symptoms

***had been on a riboflavin dose of 50 mg/kg/day for 6 months prior to increase in dose up to 60 mg/kg/day 2 months ago

**Supplementary Table 4 Response to riboflavin therapy in Patient E1**

| **Testing Modality** | **Prior to Riboflavin** | **After 3 Months of Riboflavin** |
| --- | --- | --- |
|  |  |  |
| **Audiometry** | 80db at 8kHz | 40-55db at 8kHz |
|  |  |  |
|  |  |  |
| **Visual Evoked Potentials** | Size BEO 200' degraded 100' 10uV at 176ms 50' 14uV at 175ms | Size BEO 200' 16uV at 160ms 100' 16uV at 154ms 50' 8uV at 131ms |
|  |  |  |
| **Forced Vital Capacity** | 0.51L (29%) | 0.56 L (33%) |
|  |  |  |
|  |  |  |
| **Acylcarnitine Profile** | Mimicking MADD^+^ | Normal^+^ |
|  |  |  |

^+^Complete acylcarnitine profiles included in Supplementary Table 2

BEO = both eyes open; kHz = kilohertz; L = litres; MADD = multiple acyl-CoA dehydrogenase deficiency; ms = milliseconds

| **Patient**  **Supplementary Table 5 Sural nerve pathology findings in patients with mutations in *SLC52A2*** | **E2** | **E3** | **E5** | **A3** | **I1** |
| --- | --- | --- | --- | --- | --- |
| **Sex** | Female | Female | Female | Female | Male |
| **Age at nerve biopsy** | 2 yrs | 4 years | 4 years | 3 years | 2 years |
| **Axonal loss** | 2+; LMF > SMF | 2+; LMF>SMF | 1+ LMF>SMF | 2+ LMF>SMF | 3+ LMF>SMF |
| **Fibrosis** | 2+ | 1+ | 1+ | 1+ | 3+ |
| **Degeneration** | Mild | Moderate | Moderate | Mild | Mild |
| **Regeneration** | Absent | Absent | Absent | Absent | Absent |
| **Demyelination** | Absent | Absent | Absent | Absent | Absent |
| **Endoneurial inflammation** | 0-1/F | Absent | Absent | Absent | Absent |
| **Epineurial inflammation** | Occasional single cell | Absent | Absent | Absent | Absent |
| **Endoneurial macrophages** | Sparse | Sparse | Sparse | Absent | Sparse |
| **Electron microscopy** | Confirms chronic axonal neuropathy | Confirms chronic axonal neuropathy | Confirms chronic axonal neuropathy | Not done | Confirms chronic axonal neuropathy |
| **Teased fibres** | Not done | Axonal degeneration | Axonal degeneration | Not done | Not done |
| **Morphometry** | Not done | g ratio* normal for age; LMF loss confirmed | Not done | Not done | Not done |

*g ratio = axon diameter/total nerve fibre diameter (see Supplementary Figure 1)

F = field; LMF = large myelinated fibres; SMF = small myelinated fibres

**Supplementary Figure 1: Scatterplot of g ratio measurements as a function of axon diameter***


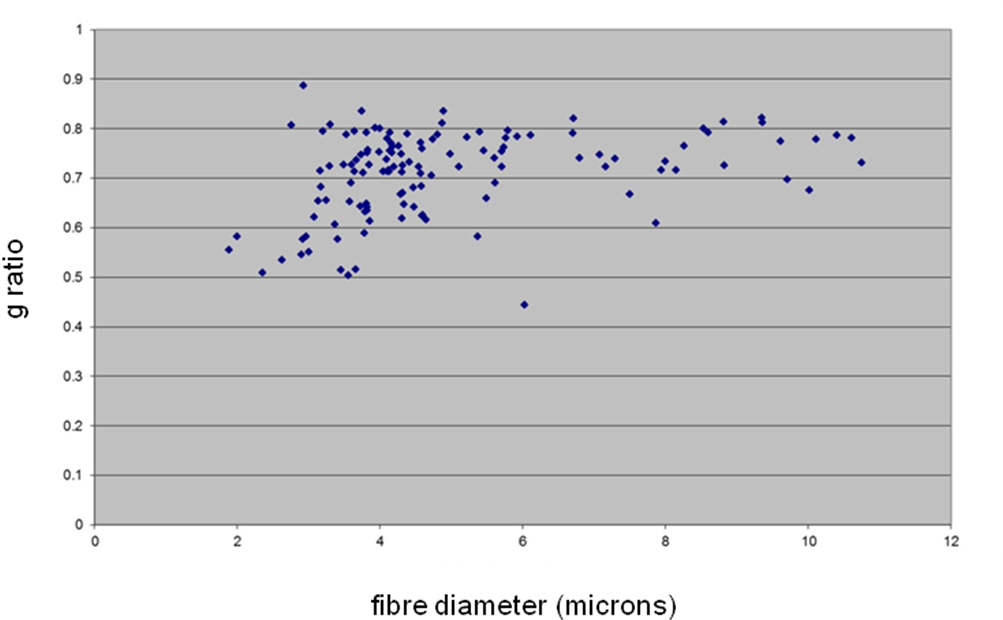


g ratio = axon diameter/total nerve fiber diameter

* as measured in the sural nerve biopsy of patient E3

Expected g-ratios at 3 years (Jacobs and Love, 1985):
0.75 - 0.85 for small calibre axons (< 7 microns)
0.65 - 0.75 for large calibre axons (> 7 microns)

In summary, while most of the surviving axons demonstrate normal morphology, there is evidence of some atrophic axons as well.

| **Supplementary Table 6 Numerical data for patients with repeat neurophysiology studies*** |  |  |  | **Family 1** | |  |
| --- | --- | --- | --- | --- | --- | --- |
| **Patient** | **E1** | **E2** | **E6** | **A1** | **A2** | **A4** |
| **Age at nerve conduction testing** | 8 yrs | 2 yrs | 2 yrs | 8 yrs | 3 yrs | 8 yrs |
| **SNAP amplitude: distal / proximal (µV)** | SNAPs absent in ULs and LLs | median: 1.47  radial: absent  sural: absent | SNAPs absent in ULs  sural: 6 | SNAPS absent in ULs and LLs | median: 2  ulnar: absent  sural: absent | median: 3  ulnar: 3  sural: absent |
| **SNAP conduction velocity (m/s)** | SNAPs absent in ULs and LLs | median: 26.9 | sural: 34 | SNAPS absent in ULs and LLs | median: not calculated | median: not calculated  ulnar: not calculated |
| **CMAP amplitude: distal / proximal (µV)** | median: 0.39 / 0.42  ulnar: 0.48 / 0.44  peroneal: absent  tibial: 0.51 / 0.67 | median: 0.10 / 0.079  ulnar: 0.16 / 0.057  peroneal: 0.48 / 0.40  tibial: 2.7 / 2.3 | tibial: 6.3 / 2.0 | peroneal: 4.3 / 4.5 | median: 11.1 / 10.7  ulnar: 6.0 / 7.7  peroneal: 3.8 / 4.0  tibial: 12.5 / 12.1 | median: 5.1 / 5.3  ulnar: 12.3 / 12.2  peroneal: 4.5 / 4.0 |
| **CMAP conduction velocity (m/s)** | median: 43.1  ulnar: 42.4  tibial: 55.9 | median: 20.2  ulnar: 47.4  peroneal: 34.1  tibial: 33.6 | tibial: 41 | peroneal: 44.8 | median: 46  ulnar: 55  peroneal: 47  tibial: 47 | median: 44  ulnar: 59  peroneal: 52 |
| **Age at repeat nerve conduction testing** | 10 yrs | 6 yrs | 10 yrs | 10 yrs | 9 yrs | 16 yrs |
| **SNAP amplitude: distal / proximal (µV)** | SNAPs absent in ULs and LLs | SNAPs absent in ULs; (LLs not tested) | SNAPs absent in UL; (LL not tested) | SNAPs absent in ULs and LLs | SNAPs absent in ULs and LLs | median: 5  sural: absent |
| **SNAP conduction velocity (m/s)** | SNAPs absent in ULs and LLs | SNAPs absent in ULs; (LLs not tested | SNAPs absent in UL; (LL not tested) | SNAPs absent in ULs and LLs | SNAPs absent in ULs and LLs | median: not calculated |
| **CMAP amplitude: distal / proximal (µV)** | median: 0.19 / 0.14  ulnar: 0.40 / 0.45 | median: absent  ulnar: absent  tibial: 0.4 | median: 0.9 / 0.5  ulnar: 0.1 / 0.1  (LL not tested) | median: 9.3 / 8.7  ulnar: 8.0 / 7.6  peroneal: 3.8 / 3.5  tibial: 8.0 / 6.4 | median: 3.1 / 3.1  ulnar: 4.6 / 4.5  peroneal: 1.4  tibial: 6.3 / 5.9 | median: absent  ulnar: absent  peroneal: 4.8 / 3.6  tibial: 13.2 / 10.4 |
| **CMAP conduction velocity (m/s)** | median: 48.0  ulnar: 46.9 | tibial: not calculated | median: 56  ulnar: 66 | median: 47  ulnar: 54  peroneal: 46  tibial: 45 | median: 43  ulnar: 50  tibial: 42 | peroneal: 44  tibial: 40 |

*prior to initiating riboflavin therapy

CMAP = compound motor action potential; LLs = lower limbs; m/s = metres per second; µV = microvolts; SNAP = sensory nerve action potential; ULs = upper limbs; yrs = years

**Supplementary Table 7 Primer sequences used in Sanger sequencing *SLC52A1*, *SLC52A2* and *SLC52A3***

| **Primer Location** | **Forward and Reverse Primers (5’-3’)** |
| --- | --- |
| *SLC52A1* Primer pair 1 | AGCATCTTTGGACCTACC; TAGGAAGGCCACAGAGTG |
| *SLC52A1* Primer pair 2 | GCCTGTGGTGGTAAAAGACC; TAGGGCACTGAGACCCTGAC |
| *SLC52A1* Primer pair 3 | CTGAGTGTAGTGGGCACAG; ACCATGGGCTGAGAACAG |
| *SLC52A1* Primer pair 4 | AGGAAGAAGAGGCTTTGC; ACACAGACACAGCACCCAC |
| *SLC52A1* Primer pair 5 | GAGCAAGTGGAGACATGAAG; AGCCTCACGATGAAGACAG |
|  |  |
| *SLC52A2* exon 2 | CAGTTCCCCTGGTCTCACC; CACCCTCTGGAAGCTCTCTG |
| *SLC52A2* exon 3.1 | GCAGGTGTGCCCAAGACT; GAAAACGCTCAAGGAAGTCG |
| *SLC52A2* exon 3.2 | ATGCTGTGCCTCGAATGTC; GCTCTTGCAGTGGTGAGGAC |
| *SLC52A2* exon 3.3 | CCACCACCATCTGTACCCAC; GAGCGAGCAGAATGTCAGG |
| *SLC52A2* exon 4 | GCTTTTCCTGCTTACCCTACG; GAGAACACGCCAAGACACAG |
| *SLC52A2* exon 5 | GTGGTCCTCGTGGTGAGC; CAGGCACTCAGGCATGG |
|  |  |
| *SLC52A3* exon 2.1 | TCACAGGAAGGGGAGTAATAAG; CCAGCACCCAGGAGGTC |
| *SLC52A3* exon 2.2 | TCCGAAGTGCCCATCATC; AGAAGGATGGAGGTGAGCAG |
| *SLC52A3* exon 3.1 | GCAGTCATTATTGCCACCTTG; AGGCCACCAGGGTATAGAT |
| *SLC52A3* exon 3.2 | ACCAGGTCACCCTCCACTC; TAGGTGCGTTTGGAATTCTG |
| *SLC52A3* exon 4 | TATGGAGACACTGGCCATCC; CCCAAGCTCTCCCAGGC |
| *SLC52A3* exon 5 | GCCCTGTGAGAGTTCTTTGC; GGCACTTGCGTTCATGATTC |

(For sequencing performed at the Department of Molecular Neuroscience and the MRC Centre for Neuromuscular Diseases, UCL Institute of Neurology, London, UK)

**Sequence traces:**


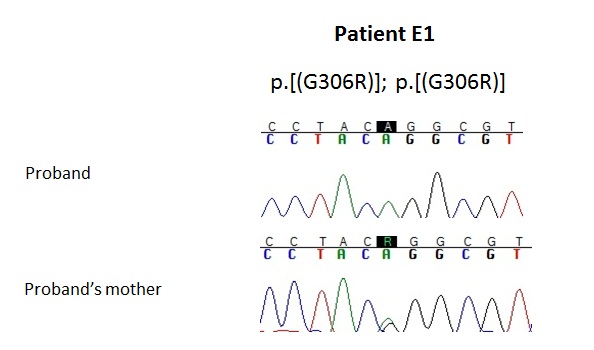

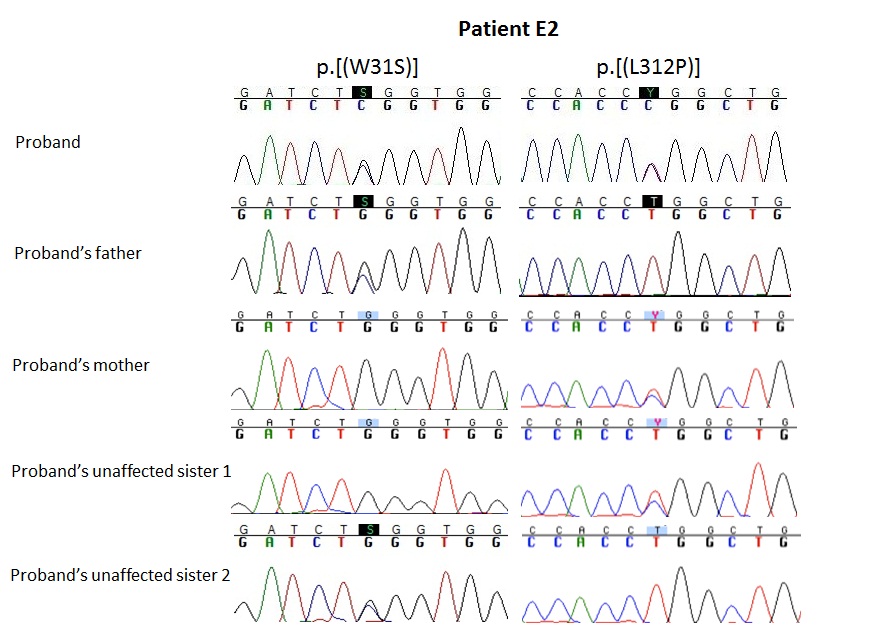

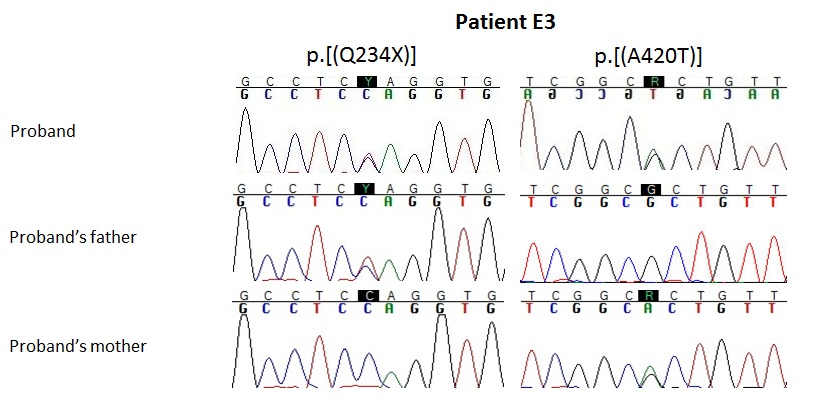

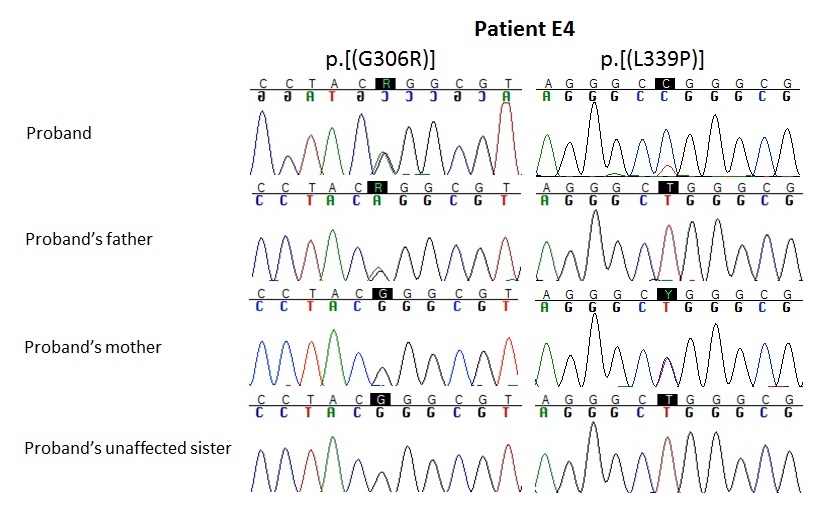

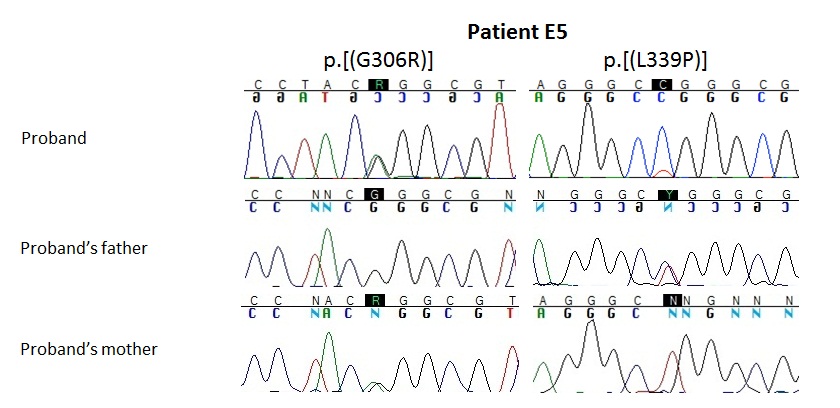

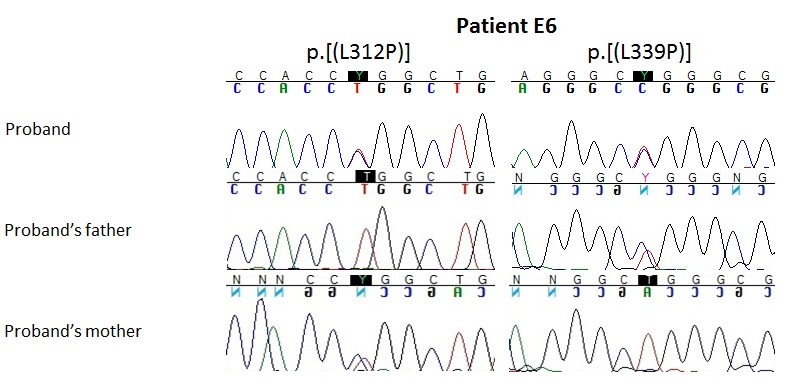

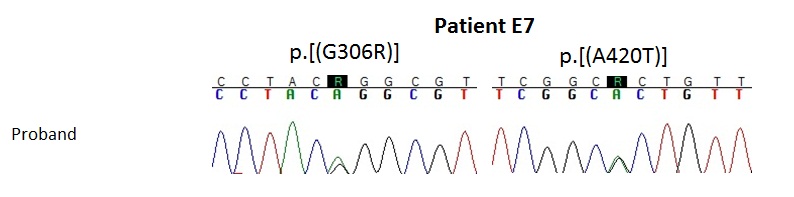

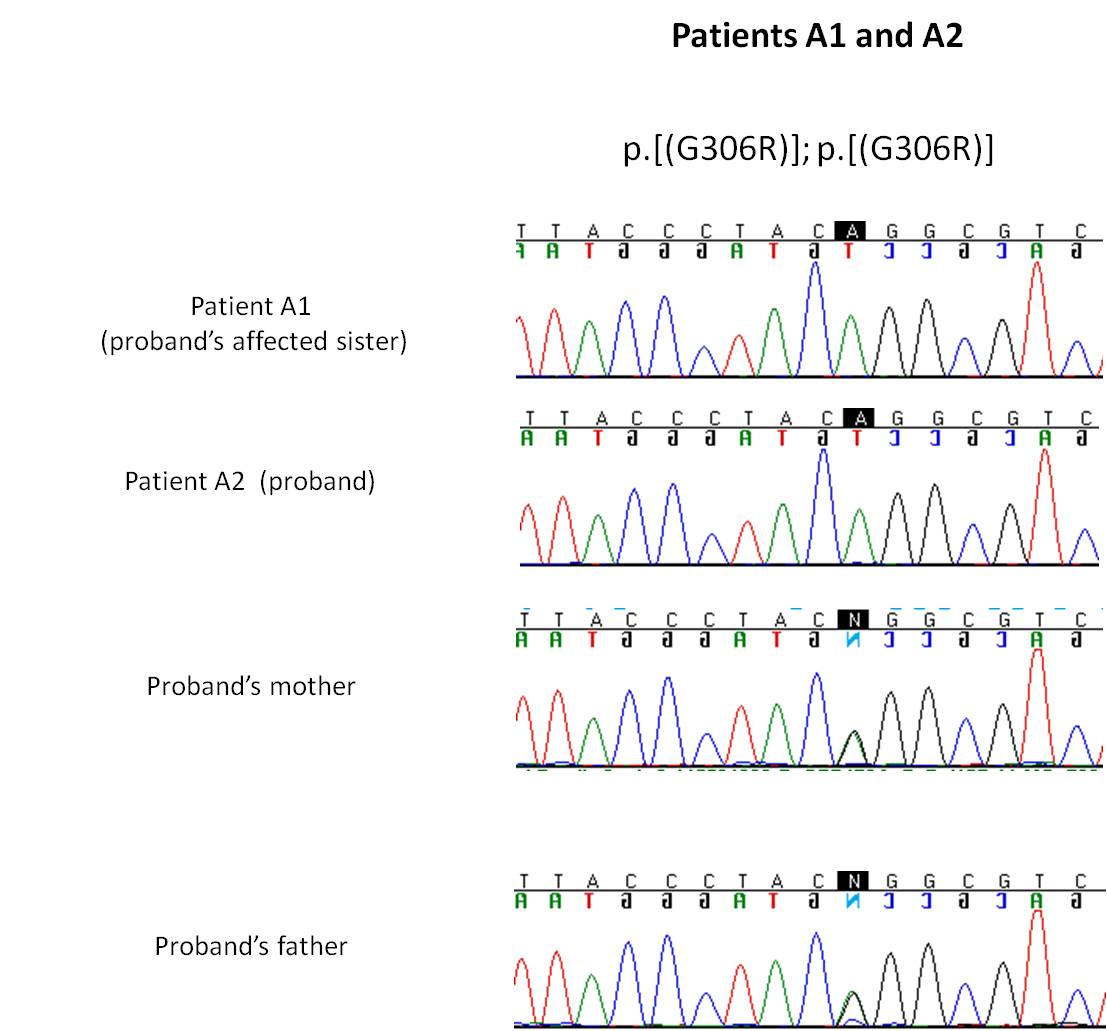

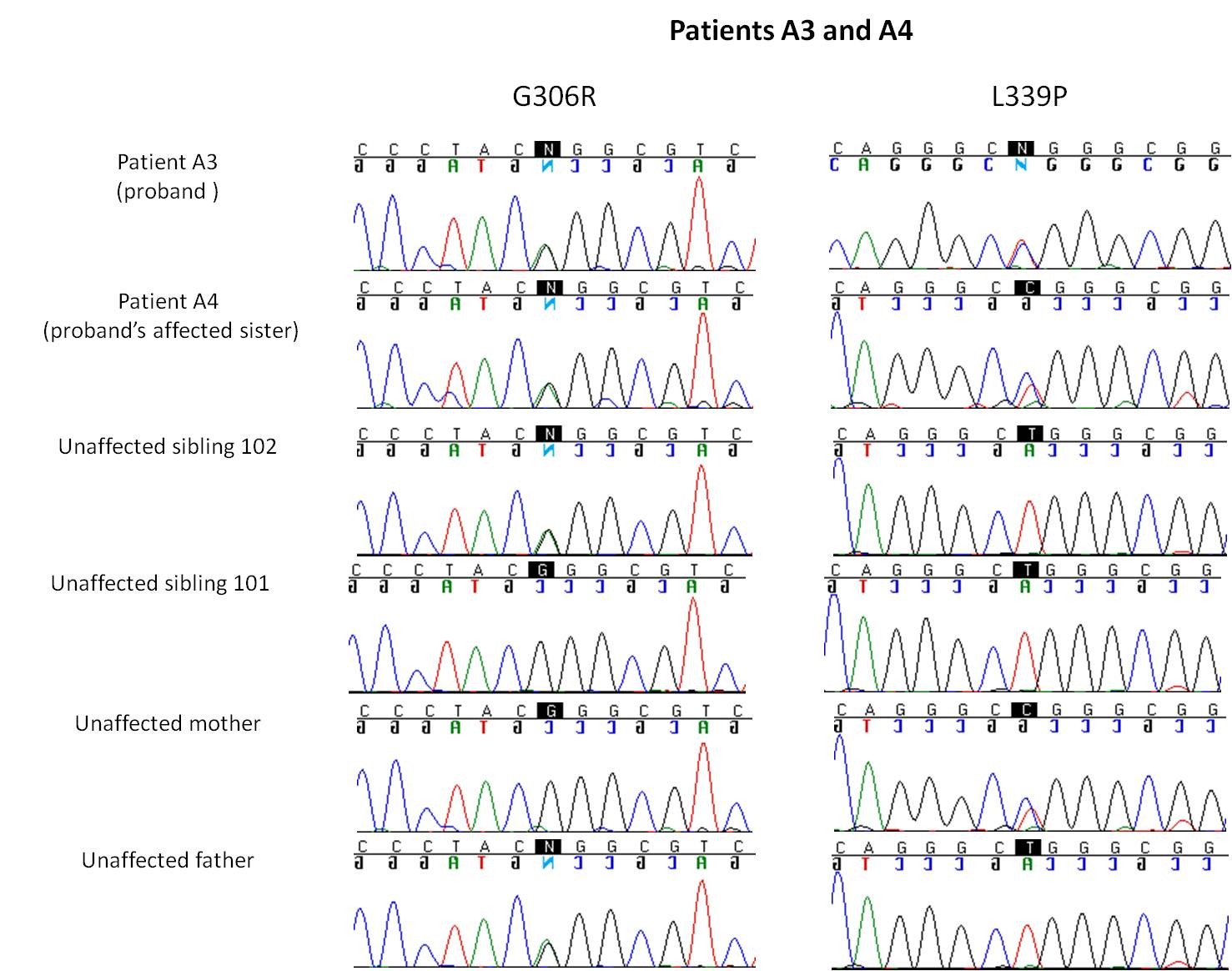

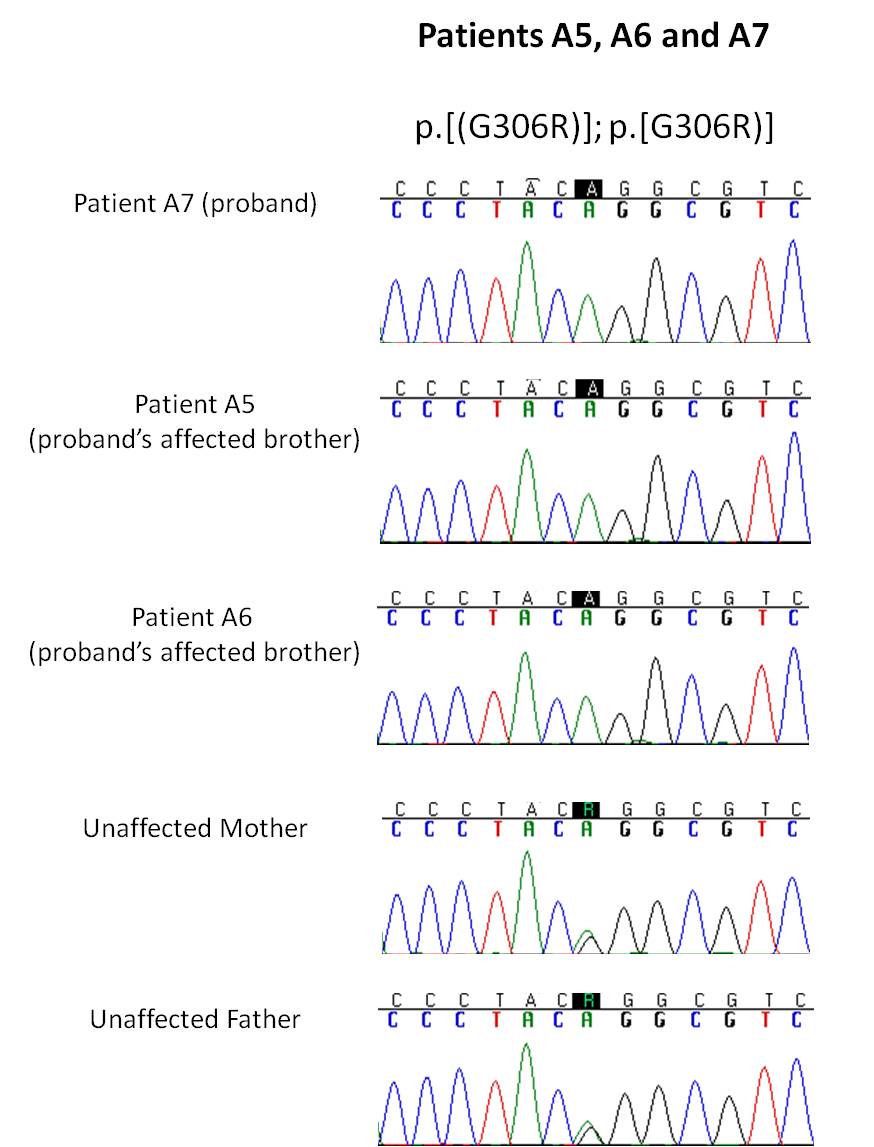

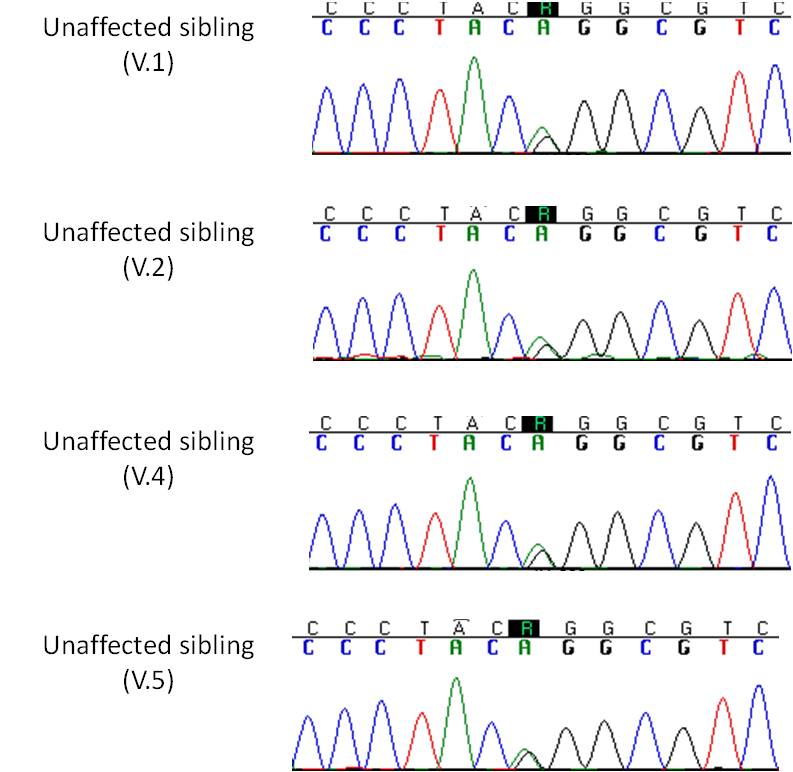

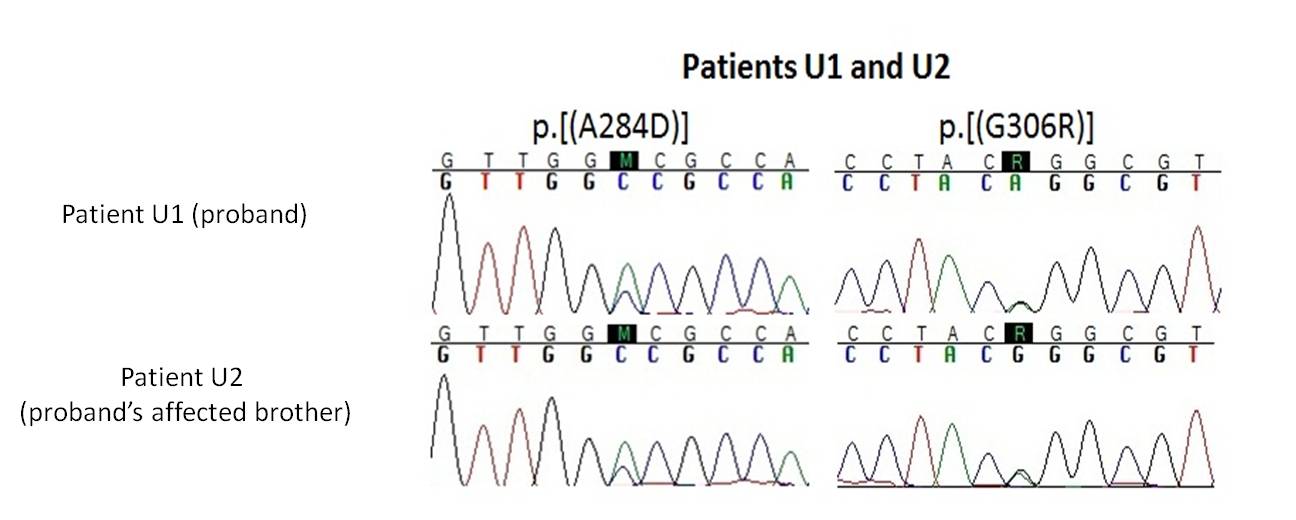

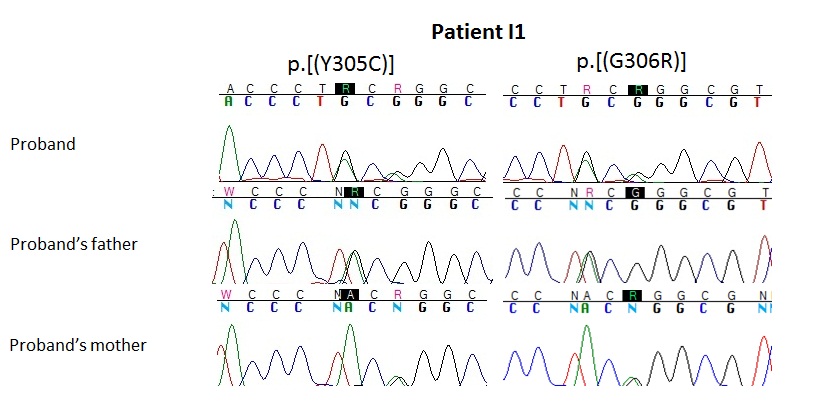

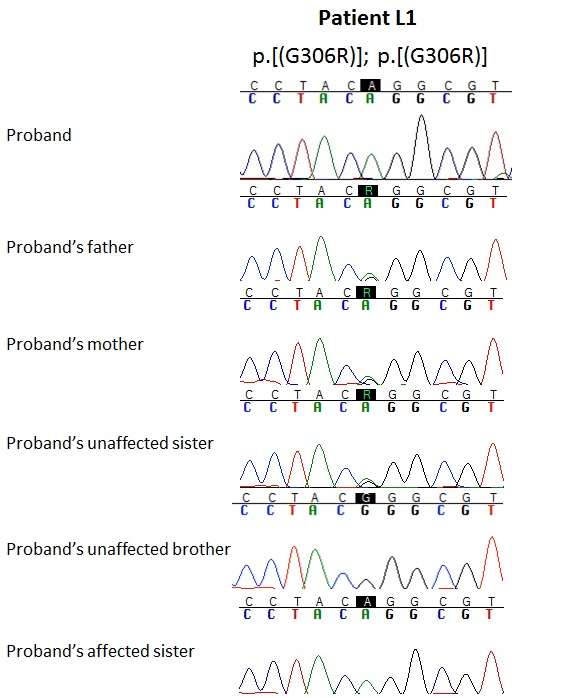


**Exome Sequencing Methods**

Exome sequencing revealed *SLC52A2* mutations in 7 patients of this cohort (A3 – A7 and U1-U2).

Exome sequencing (University of Miami)

Patients A3, A4, U1 and U2 were exome sequenced using the SureSelect Human All Exon 50MB exome capture kit (Agilent) and sequenced on the Illumina HiSeq2000 at the University of Miami. The exome sequence analysis methods are given below.

Sequence data analysis (University of Miami)

The Illumina CASAVA v1.8 pipeline was used to produce 99bp paired end sequence reads. Sequence reads were aligned to the human reference genome (hg19) using BWA (PMID: 20080505) and variations were called using the Genome Analysis ToolKit (PMID: 20644199). All variations were further characterized using the SeattleSeq134 annotation server (http://snp.gs.washington.edu/SeattleSeqAnnotation134/). The Genomes Management Application (GEMapp), University of Miami Miller School of Medicine (https://secureforms.med.miami.edu/hihg/gem-app) was used for analysis of exome variant data. Variant prioritizing included conservation scores, GERP (PMID: 15965027), PhastCons (PMID: 16024819), and minimal GATK quality scores. The GERP score quantifies position-specific evolutionary constraint in terms of rejected substitution; the neutral rate of substitution is scored 0, higher GERP scores indicate positions with higher constraint (range -11.6 to 5.82) (Cooper *et al.* , 2005). PhastCons identifies conserved elements based on a phylogenetic hidden Markov model that estimates the probability that a certain nucleotide belongs to a conserved element (range 0-1) (Siepel *et al.* , 2005). The QUAL score is the Phred probability that a variant polymorphism exists at a given site given the sequencing data, where a score of 10 indicates a 1 in 10 chance of error and a score of 100 indicates a 1 in 100 chance of error. GATK’s genotype quality score (GQ) is a Phred-scaled likelihood confidence score that the genotype of a variant is correct. For heterozygous variations, the GQ equation is as follows:


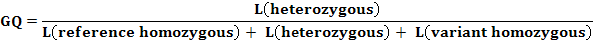


with a max score of 99. Sanger sequencing was used to analyze all variants identified and segregation carried out.

Exome sequencing (BGI)

Patients A5-A7 were exome sequenced with the NimbleGen Sequence Capture Exome Array kit (44Mb) (Roche NimbleGen) with subsequent exome sequencing on the Illumina HiSeq2000 instrument (Illumina) through BGI Inc. (http://www.genomics.cn/en/index). The exome capture procedure was performed according to the NimbleGen exome capture and sequencing protocol. The coverage of the target regions was 99.47% and the mean depth covered was 60.91%. The captured DNA was then sequenced using the Illumina HiSeq 2000 Sequencer (Illumina).

Sequence data analysis (University of Sydney)

The resulting sequencing data were mapped to the hg19 build human reference sequence using Burrows–Wheeler Aligner (BWA) (Li and Durbin, 2009). Firstly, known SNPs (variants contained in dbSNP132) were removed. Following this, a Picard tool, FixMateInformation, was used to resolve inconsistencies introduced by soft clipping in the resulting Sequence Alignment/Map (SAM) format. Picard tool, AddorReplaceReadGroups, was then used to create the Binary Alignment/Map (BAM) file (Li *et al.* , 2009), which is compatible with the Genome Analysis Tool Kit (GATK) (McKenna *et al.* , 2010). A GATK centric pipeline was utilized to perform a local realignment, base quality and variant calling recalibration. Coding variants were annotated using SNPEff (Cingolani *et al.* , 2012) which uses Ensembl/Gencode transcript annotations (ENST identifiers). In addition to Variant Quality Score Recalibration (VQSR), variants were further filtered based on function and depth (>= 10). Variants then were annotated using Annotate Variation (ANNOVAR) software (Wang *et al.* , 2010). This pipeline analysis resulted in the identification of 12 novel homozygous missense variants in the proband (A7). Sanger sequencing was used to analyze all variants identified and segregation carried out.

Sanger sequencing Methods (University of Sydney**)**

Sanger sequencing was used to confirm the novel missense variation in the *SLC52A2* gene (c.916G>A, p.Gly306Arg) in the affected individuals, unaffected individuals and their parents. The following primers were designed using Primer3 software (Skaletsky, 2000) and used to amplify the target region: forward primer 5`-GCAGAGGAAGAGGTGGAAGA-3` and reverse primer 5`-GAGCGAGCAGAATGTCAGG-3`. A PCR Express Thermal Cycler (Thermo Hybaid) was used to perform the PCR cycling. Each 20 µl PCR reaction contained 1x ImmoBuffer, 0.5U Immolase DNA Polymerase, 3.5 mM MgCl_2_ solution (all provided from Bioline Pty Ltd, Alexandria, NSW, Australia), 0.2 mM of each dNTP (Astral Scientific, Sydney, Australia), 0.5 μM of both primers, 1 M betaine (Sigma-Aldrich, Castle Hill, NSW, Australia), 2% DMSO (Sigma-Aldrich, Castle Hill, NSW, Australia) and 25 ng of genomic DNA. PCR conditions were as follows: 12 min 95 °C for the denaturation step; 35 cycles of 95°C for 30s, 64°C for 30s and 70°C for 60s, and lastly 70°C for 10 min for the final extension step. The 96-capillary 3730xl DNA Sequencer (Applied Biosystems) was used to sequence the PCR products and the sequencing data was analysed using BioEdit Sequence Alignment Editor (Hall, 1999).

**Functional Analyses of *SLC52A2* Mutations - Materials and Methods**

cDNA plasmids, SLC52A2 (92G>C; W31S), SLC52A2 (700C>T; Q234X), SLC52A2 (851C>A; A284D), SLC52A2 (914A>G; Y305C), SLC52A2 (916G>A; G306R), SLC52A2 (935T>C; L312P) and SLC52A2 (1016T>C; L339P), were constructed as described previously (Haack et al. 2012). (Primer sets used in these experiments are listed Supplementary Table 8.) The mutations were confirmed by direct sequencing. Subsequently, HEK293 cells were transfected with empty vector (Vector) and pFLAG-CMV^TM^-6a Expression Vector (Sigma-Aldrich, St. Louis, MO) containing SLC52A2^WT^ (WT) and SLC52A2 variants using Lipofectamine 2000 (Life technologies, Carlsbad, CA) according to the manufacturer’s instructions. Forty-eight hours after the transfection, the cells were used for subsequent experiments.

Uptake experiments were performed according to earlier methods (Yao et al. 2010) with some modifications. Briefly, cells were incubated with 5 nM [^3^H]riboflavin (0.814 TBq/mmol, Moravek Biochemicals, Inc., Brea, CA) for 1 min, and after washing and solubilisation, the radioactivity in aliquots was determined by liquid scintillation counting. Western blot analysis was performed as described previously (Yao et al. 2010). Crude membrane fractions were separated by sodium dodecyl sulfate polyacrylamide gel and were transferred to polyvinylidene difluoride membranes. Primary antibodies were anti-FLAG M2 antibody (Sigma-Aldrich, St. Louis, MO) and Na^+^/K^+^-ATPase antibody (Upstate Biotechnology, Lake Placid, NY). Secondary antibody was horseradish peroxidase-conjugated anti-mouse IgG (GE Healthcare Bio-Sciences, Milwaukee, WI). The bound antibody was detected on X-ray film using a Luminata^TM^ Crescendo Western HRP substrate (Millipore Corporation, Billerica, MA). Reverse transcription-PCR (RT-PCR) analysis was carried out as follows. Briefly, total RNA from HEK293 cells was reverse transcribed using High capacity RNA-to-cDNA kit (Applied Biosystems, Carlsbad, CA) and then digested with RNase H (Invitrogen). The resulting single-stranded DNA fragments were amplified with the following primer sets specific for *SLC52A2* gene, forward primer 5′-GTGGCACCATGTGGCCCCAG-3′, reverse primer 5′-CAACAGCAGCAGAAGACCCT-3′ (Haack et al. 2012).

**Supplementary Table 8 Primers used for site-directed mutagenesis**

| **Mutation** | **Forward primer (5’ to 3’)** | **Reverse primer (5’ to 3’)** |
| --- | --- | --- |
| W31S | CGGTGGAGCTACCTGTGGTGGTCAAA | AGATCCCATTGACCGCAGCCCAGGA |
| Q234X | TAGGTGGGAGCCCCAGGAGCAGAGGA | GAGGCCTGATCCTAACTCCCCTGTG |
| A284D | ACGCCACCAACGCGCTGACCAATGGC | CCAACAGGCCCAGCAGGCAGGCACT |
| Y305C | GCGGGCGTCTGGCCTACCACCTGGCT | AGGGTAAGCAGGAAAAGCTCTGCAC |
| G306R | AGGCGTCTGGCCTACCACCTGGCTGT | GTAGGGTAAGCAGGAAAAGCTCTGC |
| L312P | CGGCTGTGGTGCTGGGCAGTGCTGCC | GGTGGTAGGCCAGACGCCCGTAGGG |
| L339P | CGGGCGGCCTCTCTCTGCTGGGCGTG | GCCCTGCCAAGGACCTGCACAGCAC |

**References**

Cingolani P, Platts A, Wang LL, Coon M, Nguyen T, Wang L, et al. A program for annotating and predicting the effects of single nucleotide polymorphisms, SnpEff: SNPs in the genome of Drosophila melanogaster strain w^1118^; iso-2; iso-3. Fly. 2012; 6(2): 80-92.

Cooper GM, Stone EA, Asimenos G, Green ED, Batzoglou S, Sidow A. Distribution and intensity of constraint in mammalian genomic sequence. Genome Res. 2005; 15(7): 901-13.

Haack TB, Makowski C, Yao Y, Graf E, Hempel M, Wieland T, et al. Impaired riboflavin transport due to missense mutations in SLC52A2 causes Brown-Vialetto-Van Laere syndrome. J Inherit Metab Dis. 2012; 35(6): 943-8.

Hall TA. BioEdit: a user-friendly biological sequence alignment editor and analysis program for Windows 95/98/NT. Nucleic Acids Symposium Series. 1999; 41: 95-8.

Jacobs JM, Love S. Qualitative and quantitative morphology of human sural nerve at different ages. Brain. 1985; 108 ( Pt 4): 897-924.

Johnson JO, Gibbs JR, Megarbane A, Urtizberea JA, Hernandez DG, Foley AR, et al. Exome sequencing reveals riboflavin transporter mutations as a cause of motor neuron disease. Brain. 2012; 135(Pt 9): 2875-82.

Li H, Durbin R. Fast and accurate short read alignment with Burrows–Wheeler transform. Bioinformatics. 2009; 25(14): 1754-60.

Li H, Handsaker B, Wysoker A, Fennell T, Ruan J, Homer N, et al. The Sequence Alignment/Map format and SAMtools. Bioinformatics. 2009; 25(16): 2078-9.

McKenna A, Hanna M, Banks E, Sivachenko A, Cibulskis K, Kernytsky A, et al. The Genome Analysis Toolkit: A MapReduce framework for analyzing next-generation DNA sequencing data. Genome Research. 2010; 20(9): 1297-303.

Siepel A, Bejerano G, Pedersen JS, Hinrichs AS, Hou M, Rosenbloom K, et al. Evolutionarily conserved elements in vertebrate, insect, worm, and yeast genomes. Genome research. 2005; 15(8): 1034-50.

Skaletsky SRaHJ. Primer3 on the WWW for General Users and for Biologist Programmers. In: Krawetz S MS, editor. Bioinformatics Methods and Protocols Totowa, NJ: Humana Press; 2000. p. 365-86.

Wang K, Li M, Hakonarson H. ANNOVAR: functional annotation of genetic variants from high-throughput sequencing data. Nucleic Acids Research. 2010; 38(16): e164.

Yao Y, Yonezawa A, Yoshimatsu H, Masuda S, Katsura T, Inui K. Identification and comparative functional characterization of a new human riboflavin transporter hRFT3 expressed in the brain. J Nutr. 2010; 140(7): 1220-6.
